# Nuclear to Cytoplasmic Transport Is a Druggable Dependency in HDAC7‐driven Small Cell Lung Cancer

**DOI:** 10.1002/advs.202413445

**Published:** 2025-01-30

**Authors:** Tingting Qin, Jingya Wang, Jian Wang, Qingwu Du, Liuchun Wang, Hailin Liu, Wenting Liu, Xueyang Li, Yantao Jiang, Qi Xu, Junjie Yu, Huiyan Liu, Ting Wang, Mengjie Li, Dingzhi Huang

**Affiliations:** ^1^ Tianjin Medical University Cancer Institute and Hospital National Clinical Research Center for Cancer Key Laboratory of Cancer Prevention and Therapy Tianjin 300060; ^2^ Tianjin's Clinical Research Center for Cancer Department of Thoracic Oncology Tianjin Lung Cancer Center Tianjin Cancer Institute & Hospital Tianjin Medical University Tianjin 300060 P. R. China

**Keywords:** c‐Myc, exportin (XPO) 1, histone deacetylase 7, selinexor, small cell lung cancer

## Abstract

Immunotherapy has gained approval for use in small cell lung cancer (SCLC), yet only a subset of patients (10–20%) experience meaningful benefits, underscoring the urgent need for more effective therapeutic approaches. This work discovers a distinct HDAC7‐high SCLC phenotype characterized by enhanced proliferative potential, which recurs across various subtypes and serves as a predictor of poorer survival outcomes. By analyzing public datasets, this work finds a strong correlation between c‐Myc and HDAC7. RNA sequencing and cellular experiments show that XPO1 is a key regulator in the HDAC7/c‐Myc axis. HDAC7 promotes β‐catenin deacetylation, phosphorylation modulation, nuclear translocation, and formation of the β‐catenin/TCF/LEF1 complex, which binds to c‐Myc and XPO1 promoters. Activation of the HDAC7/β‐catenin pathway upregulates c‐Myc and XPO1 expression, while c‐Myc also boosts XPO1 expression. Given the difficulty in targeting c‐Myc directly, this work tests selinexor and vorinostat in SCLC xenograft models, with selinexor showing superior results. High HDAC7 expression is linked to increased SCLC proliferation, poorer prognosis, and enhanced sensitivity to selinexor in SCLC cell lines and organoid models. Collectively, this work uncovers a novel HDAC7/c‐Myc/XPO1 signaling axis that promotes SCLC progression, suggesting that HDAC7 may warrant further investigation as a potential biomarker for assessing selinexor sensitivity in SCLC patients.

## Graphical abstract

This work reports that a HDAC7‐high SCLC subtype is associated with enhanced proliferation and a poor prognosis. Furthermore, nuclear‐to‐cytoplasmic transport is emerged as a druggable dependency in HDAC7‐mediated SCLC. Elevated HDAC7 predicts increase sensitivity to selinexor both in vitro and in patient‐derived organoids. Consequently, this work proposes selinexor as a promising therapeutic option for SCLC patients exhibiting high HDAC7 expression.

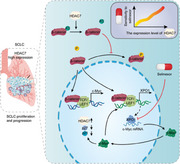

## 1 Introduction

Small cell lung cancer (SCLC) accounts for ≈15% of lung cancers, and is highly aggressive with limited treatment options and poor prognosis, with a 5‐year survival rate <6%.^[^ ^1^ ^]^ The incorporation of immunotherapy into first‐line platinum‐etoposide chemotherapy has only modestly enhanced outcomes for SCLC patients in unselected populations.^[^ ^2^ ^]^ Regrettably, targeted therapies have shown limited to no success in SCLC, failing to achieve the desired results, including those targeting poly(ADP‐ribose) polymerase (PARP)^[^ ^3^ ^]^ and B‐cell lymphoma‐2 (BCL2).^[^ ^4^ ^]^ Compared to non‐small cell lung cancer (NSCLC), SCLC exhibits greater tumor diversity, driven by canonical, intermediate, and mixed subtypes.^[^ ^5^ ^]^ Despite the identification of numerous SCLC molecular subtypes,^[^ ^6^ ^]^ they have not significantly advanced or altered the targeted therapy landscape for this disease.^[^ ^7^ ^]^ Consequently, the need for personalized treatment strategies is evident, and the pursuit of individualized molecular therapies is gaining significant attention.^[^ ^8^ ^]^ Our groundbreaking research was the first to unveil exportin 1 (XPO1), a pivotal regulator of nuclear export for various proteins and mRNAs, as a promising novel therapeutic target for SCLC.^[^ ^9^ ^]^ Subsequent laboratory findings by Rudin's team further validated our discovery, demonstrating that XPO1 inhibition enhances SCLC's sensitivity to chemotherapy^[^ ^10^ ^]^ and halts its neuroendocrine transformation.^[^ ^11^ ^]^ These results highlight the promising potential of XPO1 as a therapeutic target in SCLC. Despite these advancements, numerous past studies on targeted therapies have emphasized that efficacy can vary significantly based on an individual's genetic profile, even when targeting the same molecular entity. Specifically, EGFR co‐mutations have been associated with reduced responses to tryosine kinase inhibitors (TKIs),^[^ ^12^ ^]^ while high programmed cell death‐ligand 1 (PD‐L1) expression has similarly been correlated with suboptimal TKI efficacy.^[^ ^13^ ^]^ Even with selinexor, an XPO1 inhibitor initially approved for diffuse large B‐cell lymphoma (DLBCL) and multiple myeloma (MM), the objective response rates when used as monotherapy or in combination with dexamethasone were 25%^[^ ^14^ ^]^ and 28%,^[^ ^15^ ^]^ respectively. This indicates that there is still room for improving efficacy, and careful patient selection remains crucial. Furthermore, the genetic characteristics of the potential beneficial patient population for XPO1 inhibitors in SCLC remain largely unknown and require further elucidation.

Despite a >60% response rate to cytotoxic therapies, most patients with SCLC rapidly relapse.^[^ ^1^ ^]^ Cancer cells are able to counteract chemotherapeutic effects through epigenetic modifications, particularly abnormal alterations to genomic DNA or histone proteins.^[^ ^16^ ^]^ Histone deacetylase enzymes (HDACs) play a pivotal role in mediating the post‐translational acetylation of various histone proteins, which serve as essential mediators in the modulation of gene expression. HDACs control numerous cancer‐related processes, including cell proliferation, migration, apoptosis, and angiogenesis. Recent research has shown that abnormal expression of HDACs is associated with tumor initiation and progression.^[^ ^17^ ^]^ Within the HDAC family, which is divided into four classes, the HDAC IIa subgroup comprises HDAC4, HDAC5, HDAC7, and HDAC9. This subgroup has several vital functions in immunological and neurological disorders, diabetes, and muscle degenerative diseases.^[^ ^18^ ^]^ The role of these proteins in cancer has garnered significant attention. For instance, inhibiting HDAC4 enhances breast cancer metastasis by increasing NEDD9 expression.^[^ ^19^ ^]^ HDAC5 modulates PD‐L1 expression and cancer immunity via p65 deacetylation in pancreatic cancer.^[^ ^20^ ^]^ HDAC9 is indispensable for the proliferation of KMT2A‐rearranged acute myeloid leukemia.^[^ ^21^ ^]^ HDAC7 is essential in the critical pathological processes of angiogenesis^[^ ^22^ ^]^ and in immunity.^[^ ^23^ ^]^ Accumulating evidence suggests that HDAC7 also plays a significant role in the development of various diseases, including cancer,^[^ ^24^ ^]^ HDAC7 overexpression has been implicated in driving tumor progression across various cancer types, including esophageal squamous cell carcinoma (ESCC),^[^ ^25^ ^]^ NSCLC,^[^ ^26^ ^]^ and breast and ovarian cancer.^[^ ^27^ ^]^ Conversely, HDAC7 loss or misregulation may lead to B‐cell‐based hematological malignancies.^[^ ^28^ ^]^ However, it remains unknown whether HDAC7 is involved in SCLC progression.

Recent studies have revealed that oncogenic actions of HDAC7 are reliant on the amplification of c‐Myc.^[^ ^25^, ^29^ ^]^ c‐Myc is a pivotal transcription factor capable of regulating the expression of numerous genes in cancer cells.^[^ ^30^ ^]^ Overexpression of c‐Myc is believed to be a key driver in the development of many human cancers.^[^ ^31^ ^]^ In conditional transgenic mouse cancer models, inactivation of c‐Myc leads to sustained tumor regression.^[^ ^32^ ^]^ Therapeutic targeting of c‐Myc has the potential for a broad clinical impact across various types of human cancer. However, the undruggability of c‐Myc, combined with the challenges in identifying small molecules that can directly target it, presents a significant hurdle in cancer therapy.

**Scheme 1:**
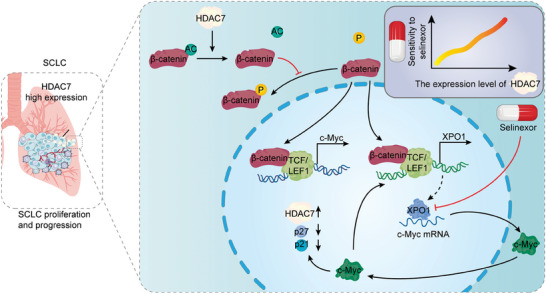
HDAC7 level predicts selinexor sensitivity in SCLC progression mechanism.

In this study, we have pinpointed a distinct subtype of SCLC characterized by elevated HDAC7 expression. This subtype encompasses the entire diversity of SCLC subtypes and is associated with poorer survival outcomes. Our findings reveal that HDAC7 functions as an oncogenic protein in SCLC by directly enhancing the expression of XPO1 and c‐Myc. Furthermore, we demonstrated that HDAC7 initiates a cascade of events, first deacetylating β‐catenin at Lys49 and subsequently dephosphorylating it at Ser552. This modification facilitates the nuclear translocation of β‐catenin, leading to the formation of a β‐catenin/TCF/LEF1 complex that binds to the promoter regions of XPO1 and c‐Myc. Notably, we discovered two c‐Myc binding sites within the XPO1 promoter, confirming that c‐Myc, in addition to HDAC7, can directly modulate XPO1 expression. Our in‐depth analysis also uncovered a complex interplay between HDAC7, XPO1, and c‐Myc in SCLC, where they mutually regulate each other. Based on the compelling evidence from xenograft mouse models and patient‐derived organoids (PDOs), we suggest that selinexor holds therapeutic promise for patients with HDAC7‐positive SCLC subtypes. Importantly, we have established that the HDAC7/XPO1/c‐Myc axis drives SCLC progression and, specifically, identified XPO1 as a highly promising therapeutic target in HDAC7‐mediated SCLC (**Scheme** **1**).

A schematic illustration outlines the mechanism of HDAC7‐mediated tumor progression, with the level of HDAC7 serving as a predictor of sensitivity to selinexor treatment in SCLC.

## 2 Results

### 2.1 Elevated HDAC7 Expression Correlates with SCLC Subtypes and Escalated Disease Progression

**Figure 1:**
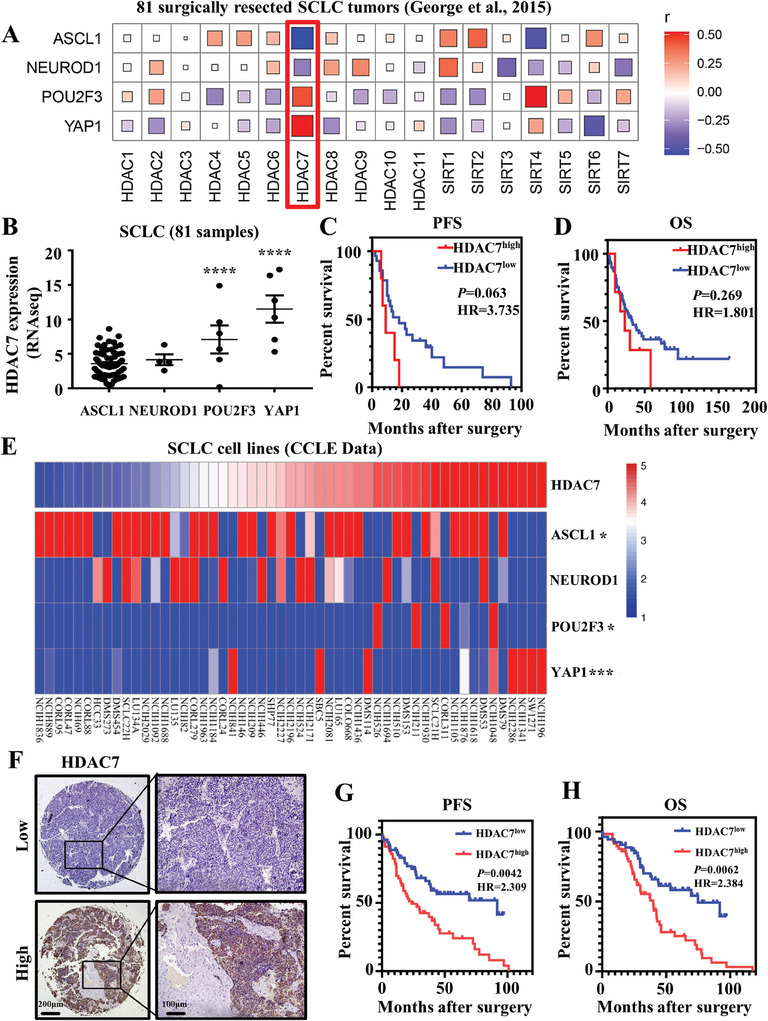
Elevated HDAC7 expression correlates with SCLC subtypes and escalated disease progression. A) Spearman correlation analysis between the mRNA expression of HDAC families and four key transcription factors of SCLC based on resected SCLCs (*n* = 81; George et al.). The size of the square indicates the degree of correlation. B) Dot plot showing HDAC7 expression among each subtype in primary human tumors (*n* = 81; George et al.). Data are the mean ± SD. *** *p* < 0.001, one‐way ANOVA. C) Kaplan–Meier plots of PFS rate of patients with SCLC exhibiting high (Red) or low (Blue) HDAC7 levels (*p* = 0.063, log‐rank test). D) Kaplan–Meier plots of OS rate of patients with SCLC exhibiting high (Red) or low (Blue) HDAC7 levels (*p* = 0.269, log‐rank test). E) Heatmap showing relative mRNA expression of HDAC7 across multiomics subtypes based on SCLC cell lines in CCLE (*n* = 54). F) HDAC7 immunostaining of specimens from patients exhibiting different HDAC7 levels; Brown, HDAC7; Blue, nuclei; Left, Scale bar, 200 µm; Right, Scale bar, 100 µm. G) Kaplan–Meier plots of PFS rate of patients with SCLC exhibiting high (Red, H‐score ≥ 120) or low (Blue, H‐score <120) HDAC7 levels (*p* = 0.0042, log‐rank test). H) Kaplan–Meier plots of OS of patients with SCLC exhibiting high (Red, H‐score ≥ 120) or low (Blue, H‐score < 120) HDAC7 levels (*p* = 0.0062, log‐rank test).

To identify novel features of HDACs involved in SCLC progression, we analyzed previous mRNA‐sequencing profiles from 81 surgically resected, mostly limited‐stage SCLC.^[^ ^33^ ^]^ We conducted a spearman correlation analysis between the mRNA expression of 18 HDAC family members and four key transcription factors of SCLC in George cohorts. The size of the square indicates the degree of correlation. Among all HDAC families, HDAC7 showed the highest correlation with SCLC subtypes (Correlation coefficient: ASCL1: ‐0.55, NEUROD1: ‐0.32, POU2F3: 0.44, YAP1: 0.52; all *p* < 0.001) (**Figure** **1**A). Next, we utilized the RNA sequencing data from these 81 SCLC cases to analyze the expression levels of HDAC7 across the four subtypes. We found that *HDAC7* mRNA was highly expressed in the SCLC‐P and SCLC‐I groups, while SCLC‐A and SCLC‐N had a comparable low level of *HDAC7* expression (Figure 1B). Patients with high HDAC7 expression exhibit worse progression‐free survival (PFS) (Figure 1C) and overall survival (OS) (Figure 1D), although the *P*‐value did not reach statistical significance, hazard ratio (HR) values was 3.735 and 1.801, respectively. These data suggested that high *HDAC7* expression was a risk factor in the 81 surgically resected SCLCs. We further analyzed mRNA expression level among 54 SCLC cell lines from the Cancer Cell Line Encyclopedia (CCLE). The YAP1 group (SCLC‐I) was positively correlated with *HDAC7* expression (*p* < 0.005), followed by the POU2F3 group (*p* < 0.05). NEUROD1 expression level showed no correlation with *HDAC7* (*p* > 0.05). ASCL1 showed negative correlation with *HDAC7* (*p* < 0.05) (Figure 1E). These results were largely consistent with the data from resected SCLC (Figure 1B).

The prognostic role of HDAC7 expression in SCLC has not been reported. To verify the role of HDAC7 in SCLC, we compared HDAC7 expression patterns in 110 clinical specimens of SCLC by immunohistochemistry (IHC) analysis (Figure 1F; Table S4, Supporting Information). In addition, SCLC patients with high HDAC7 expression (H‐score ≥ 120; n = 57) had poorer PFS compared with patients with low HDAC7 expression (*p* = 0.0042; HR = 2.309) (Figure 1G). OS was also markedly worse for patients with SCLC with high HDAC7 expression than for those with low HDAC7 expression (*p* = 0.0062; HR = 2.384) (Figure 1H). There were no statistically significant differences between HDAC7 low and high groups with regard to gender, age, smoking history, tumor size, T stage, distant metastasis, and postoperative therapy, although TNM stage and lymphatic invasion did differ significantly (Table S5, Supporting Information). Univariate and multivariate analyses identified HDAC7 (high/low), lymphatic metastasis, and TNM stage as independent prognostic factors in SCLC patients (Table S6, Supporting Information). These findings indicate that elevated HDAC7 expression in SCLC is strongly correlated with SCLC subgroups and facilitates disease progression.

### 2.2 Decreasing HDAC7 Expression Levels in Cancer Cells Leads to Slowed SCLC Cell Proliferation and Suppressed Tumor Progression

**Figure 2:**
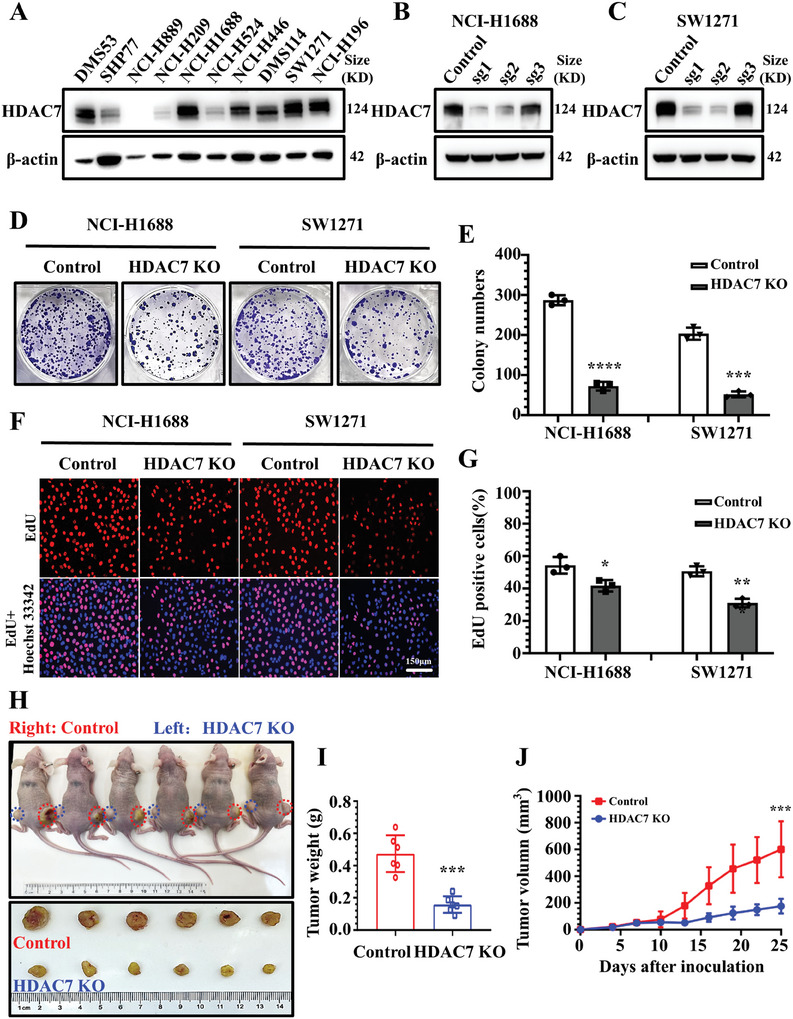
Decreasing HDAC7 expression levels in cancer cells leads to slowed SCLC cell proliferation and suppressed tumor progression. A) Western blotting showing HDAC7 protein levels in different subtypes of SCLC cell lines. B) Western blotting analysis shows HDAC7 protein levels in NCI‐H1688 cells treated with lentiCRISPR v2 and a control or sgRNAs targeting HDAC7. C) Western blotting analysis showing HDAC7 protein levels in SW1271 cells treated with lentiCRISPR v2 and a control or sgRNAs targeting HDAC7. D) Colony formation assays in SCLC cell lines with or without HDCA7 KO; Blue, colony. E) Changes in the numbers of colonies in SCLC cell lines with or without HDCA7 KO; Data are the mean ± SD. *** *p* < 0.005, **** *p* < 0.005, Student's *t*‐test. F) Typical fluorescent confocal microscopic images of EdU‐positive cells in SCLC cell lines with or without HDCA7 KO; Red, Edu positive cells; Blue, nuclei; Scale bar, 150 µm. G) Percentages of SCLC cells positive for EdU with or without HDCA7 KO; Data are the mean ± SD. * *p* < 0.05, Student's *t*‐test. H) Representative images showing xenograft tumors in each group following subcutaneously injection of NCI‐H1688 cells with or without HDAC7 KO; Ruler unit: cm. I) Analysis of tumor weight in NCI‐H1688‐HDAC7 control or ‐HDAC7 KO tumor (n = 6); Data are the mean ± SD. *** *p* <0.005, Student's *t*‐test. J) Tumor growth in mice injected with 5 × 10^6^ stable NCI‐H1688 cell lines with or without HDAC7 KO (*n* = 6); Data are the mean ± SD. *** *p* < 0.005, Student's *t*‐test.

To investigate the role of HDAC7 in SCLC proliferation and malignant progression, we compared HDAC7 protein level in different SCLC cell lines. HDAC7 was highly expressed (100%) in all three cells lines of SCLC‐I subtype, such as DMS114, SW1271, and NCI‐H196. In contrast, ratio of HDAC7 high expression (DMS53 and NCI‐H1688) was lower (42.8%) in cell lines of SCLC‐A subtype, and NCI‐H446 cell line of SCLC‐N subtype (**Figure** **2**A). Despite the varying HDAC7 positivity rates among different subtypes, we can observe that high expression of HDAC7 spans across cell lines of various subtypes, consistent with the analysis results from CCLE. Based on the basal HDAC7 levels of SCLC cell lines, we generated two stable HDAC7 knockout (KO) cell lines using NCI‐H1688 and SW1271 by CRISP/Cas9 nuclease system (Figure 2B and C). Compared with the control groups, HDAC7 KO cells had markedly decreased cell clonogenic ability, as detected by colony formation assay (Figure 2D). Quantitative analysis showed that the control NCI‐H1688 and SW1271 cells had 280 and 200 colonies, respectively, compared with only 70 and 40 in the NCI‐H1688 and SW1271 KO cells (Figure 2E) The 5‐ethynyl‐2‐deoxyuridine (EdU) incorporation assay showed fewer positive cells in the HDAC7 KO group than in the control group (Figure 2F). Quantitative measurement confirmed an ≈23% and ≈33% decrease in EdU positivity rate in the HDAC7 KO group compared with the control group of NCI‐H16688 and SW1271 cells (Figure 2G).

We examined whether HDAC7 functioned in vivo. We established the NCI‐H1688 cell xenograft in athymic nude mice, and HDAC7 KO cells resulted in significantly reduced tumor growth (Figure 2H). Median tumor weight was approximately ≈2.2‐fold lower in the HDAC7 KO group than in the control group (Figure 2I). The HDAC7 KO group showed a marked inhibition in primary tumor growth (Figure 2J). These data indicate that increased HDAC7 expression levels in cancer cells leads to SCLC cell proliferation and tumor progression.

### 2.3 c‐Myc Emerges as a Potential Gene Implicated in HDAC7‐Mediated Tumor Progression in SCLC

**Figure 3:**
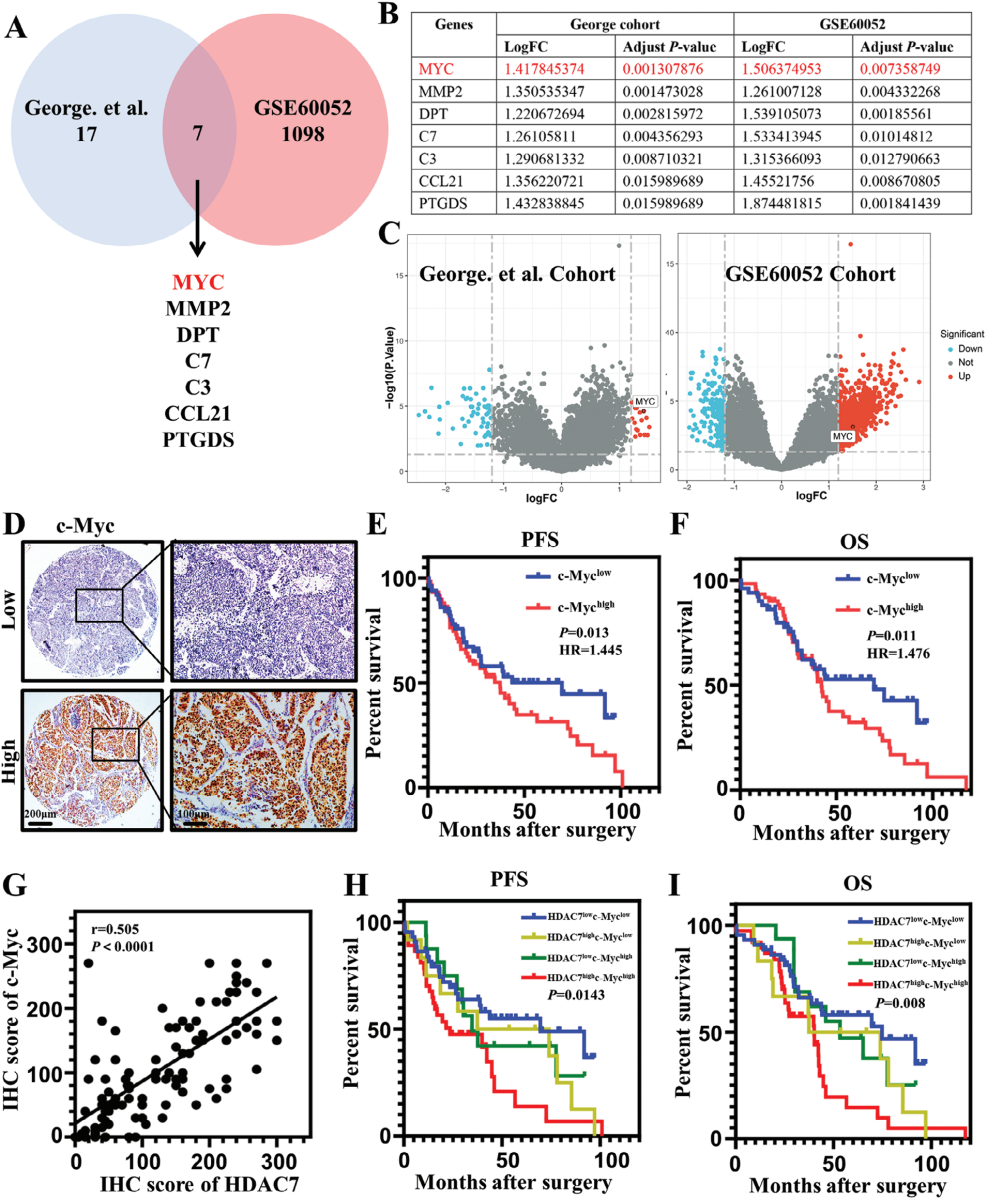
c‐Myc emerges as a potential gene implicated in HDAC7‐mediated tumor progression in SCLC. A) Venn diagram showing overlap of upregulated genes between HDAC7 high and HDAC7 low group from the George et al. cohort and GSE60052. B) Expression level of seven candidate genes. C) Volcano plot illustrating that c‐Myc was significantly altered between patients with low and high HDAC7 expression. D) Typical images of c‐Myc immunostaining in cross‐sections of SCLC patients. Brown, c‐Myc; Blue, nuclei. Left, Scale bar, 200 µm; Right, Scale bar, 100 µm. E) Kaplan–Meier plots of PFS rate of patients with SCLC exhibiting high (Red, H‐score ≥ 90) or low (Blue, H‐score < 90) c‐Myc levels (*p* = 0.013, log‐rank test). F) Kaplan–Meier plots of OS of patients with SCLC exhibiting high (Red, H‐score ≥ 90) or low (Blue, H‐score < 90) c‐Myc levels (*p* = 0.011, log‐rank test). G) Scatterplot showing Spearman's correlation of IHC score of c‐Myc and HDAC7 (*r* = 0.505, *p* < 0.0001). H) Kaplan–Meier analysis of association between PFS and expression of c‐Myc and HDAC7 in SCLC patients. I) Kaplan–Meier analysis of association between OS and the expression of c‐Myc and HDAC7 in SCLC patients.

To identify novel mechanisms underlying HDAC7‐mediated SCLC progression, we analyzed the mRNA‐sequencing profiles of SCLC patients with low and high *HDAC7* expression. RNA‐sequencing data were obtained from the George et al. cohort^[^ ^33^ ^]^ and GSE60052.^[^ ^34^ ^]^ The criteria were log fold change >1.2 and false discovery rate <0.05. The Venn diagram showed that the most significant cross‐upregulated genes were *MYC* (also known as *c‐Myc*), *MMP2*, *DPT*, *C7*, *C3*, *CCL21*, and *PTGDS* (**Figure** **3**A). Among these genes, *c‐Myc* had the most significant change and the smallest *p* value when considering these two datasets together (Figure 3B). The volcano plot showed that *c‐Myc* was one of the top‐ranked differentially upregulated genes in both datasets (Figure 3C). To evaluate further the clinical relevance of c‐Myc and HDAC7 expression in SCLC tissues, IHC was applied to detect c‐Myc level (Figure 3D). SCLC patients with high c‐Myc expression exhibited poorer PFS rate compared with patients with low c‐Myc expression (*p* = 0.013; HR = 1.445) (Figure 3E). OS was also markedly worse for patients with SCLC with high c‐Myc expression than for those with low c‐Myc expression (*p* = 0.011; HR = 1.476) (Figure 3F). We found a positive correlation between HDAC7 IHC scores and c‐Myc IHC scores (Spearman r = 0.505, *p* < 0.0001) (Figure 3G). Patients were divided into four groups according to HDAC7 and c‐Myc levels. Kaplan–Meier analyses suggested that patients with high HDAC7/c‐Myc had the worst PFS (*p* = 0.0143) (Figure 3H) and OS (*p* = 0.008) (Figure 3I), whereas SCLC with low HDAC7/c‐Myc showed the best outcome. Therefore, these results confirmed that high levels of HDAC7 are closely related to the significant increase of c‐Myc and the prognosis of patients in clinical settings, and suggested that HDAC7 may promote the progression of SCLC through c‐Myc.

### 2.4 XPO1 is Indispensable for HDAC7/c‐Myc‐Induced Tumor Progression in SCLC

**Figure 4:**
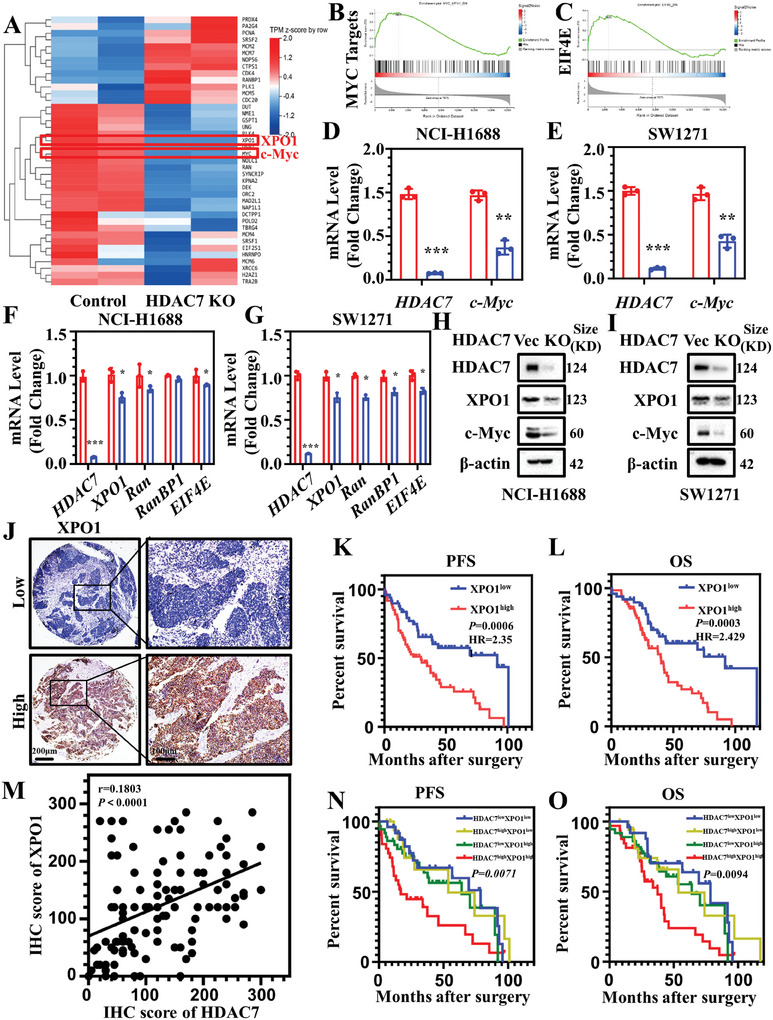
XPO1 is indispensable for HDAC7/c‐Myc‐induced tumor progression in SCLC. A) Heatmap illustrating differential expression of Myc target gene signatures between stable HDAC7 control and HDAC7 KO cell lines. B,C) Gene set enrichment analysis showing that Myc targets and EIF4E pathway were enriched between stable HDAC7 control and HDAC7 KO cell lines. D) Changes in HDAC7 and c‐Myc mRNA levels in NCI‐H1688 cell line with or without HDAC7 KO. E) Levels of HDAC7 and c‐Myc mRNA in HDAC7 control and HDAC7 KO SW1271 cells lines. F) Expression of HDAC7, XPO1, Ran, RanBP1, and EIF4D in NCI‐H1688 cells with or without HDAC7 KO was evaluated by RT‐PCR. G) Levels of HDAC7, XPO1, Ran, RanBP1, and EIF4D mRNA in HDAC7 control and HDAC7 KO SW1271 cells lines. H) Western blotting of protein levels of HDAC7, XPO1, and c‐Myc in HDAC7 control and KO NCI‐H1688 cells. I) Western blotting analysis of HDAC7, XPO1, and c‐Myc in HDAC7 control and KO SW1271 cells. J) Typical images of XPO1 immunostaining in cross‐sections of SCLC patients. Brown, XPO1; Blue, nuclei. Left, Scale bar, 200 µm; Right, Scale bar, 100 µm. K) Kaplan–Meier plots of PFS rate of patients with SCLC exhibiting high (Red, H‐score ≥ 120) or low (Blue, H‐score < 120) XPO1 levels (*p* = 0.0006, log‐rank test). L) Kaplan–Meier plots of OS of patients exhibiting high (Red, H‐score ≥ 120) or low (Blue, H‐score < 120) XPO1 levels (*p* = 0.0003, log‐rank test). M) Scatterplot showing Spearman's correlation of IHC score of XPO1 and HDAC7 (*r* = 0.1803, *p* < 0.0001). N) Kaplan–Meier analysis of the association between PFS and the expression of XPO1 and HDAC7 in SCLC patients. O) Kaplan–Meier analysis of association between OS and expression of XPO1 and HDAC7 in SCLC patients. The experiments were repeated twice. Data are the mean ± SD. *** *p* < 0.001, one‐way ANOVA.

To further investigate the molecular effects of HDAC7, we conducted gene expression analysis by RNA‐sequencing in HDAC7 KO and control cell lines of NCI‐H1688. *XPO1* and *c‐Myc* are two of the top‐ranked differentially expressed genes (DEGs) among the RNA‐sequencing data (**Figure** **4**A). Gene set enrichment analysis showed significant enrichment of MYC target genes, which included transcripts associated with XPO1 functions such as *c‐Myc* RNA translocation from nucleus to cytoplasm (Figure 4B). The eukaryotic translation initiation factor (EIF)4E pathway was also enriched (Figure 4C), which was associated with mRNA processing and protein biosynthesis. That also included transcripts associated with XPO1 functions, such as RAN and RANBP1, suggesting that HDAC7 affected XPO1‐mediated cellular functions. Quantitative polymerase chain reaction (PCR) confirmed that HDAC7 KO resulted in significant reduction of *c‐Myc* mRNA in NCI‐H1688 (Figure 4D) and SW1271 (Figure 4E) cell lines. Quantitative PCR also confirmed that HDAC7 KO had an effect on *XPO1*, *RAN*, *RANBP1*, and *EIF4E* mRNA in NCI‐H1688 (Figure 4F) and SW1271 (Figure 4G) cell lines. We cultured SCLC cell lines with or without HDAC7 KO and found a significant reduction in XPO1 and c‐Myc proteins compared with in NCI‐H1688 (Figure 4H) and SW1271 (Figure 4I) control cells. These results suggested that XPO1 played a crucial role in HDAC7/c‐Myc‐mediated tumor progression.

To evaluate the clinical importance of the correlation of HDAC7 and XPO1 in SCLC, we detected XPO1 expression using IHC (Figure 4J). SCLC patients with high XPO1 expression had poorer PFS rates than patients with low XPO1 expression (*p* = 0.0006; HR = 2.35) (Figure 4K). OS was also markedly worse in patients with SCLC with high XPO1 expression than in those with low XPO1 expression (*p* = 0.0003; HR = 2.429) (Figure 4L). Additionally, we found a clear positive correlation between HDAC7 and XPO1 IHC scores (Spearman r = 0.1803, *p* < 0.0001) (Figure 4M). Patients were divided into four groups according to HDAC7 and XPO1 protein expression levels. Kaplan–Meier analysis suggested that patients with high HDAC7/XPO1 had the worst PFS (*p* = 0.0071) (Figure 4N) and OS (*p* = 0.0094) (Figure 4O), whereas SCLC patients with low HDAC7/XPO1 showed the best outcome.

We further evaluated the possible correlation between XPO1 and c‐Myc expression and found that XPO1 was positively correlated with c‐Myc (Spearman r = 0.2680, *p* < 0.0001) (Figure S1A, Supporting Information). Kaplan–Meier analysis suggested that patients with high XPO1/c‐Myc had the worst PFS (*p* = 0.0054) (Figure S1B, Supporting Information) and OS (*p* = 0.0085) (Figure S1C, Supporting Information), whereas SCLC patients with low XPO1/c‐Myc showed the best outcome. These results suggested that high HDAC7 levels are strongly correlated with markedly elevated expression of c‐Myc and its transfer protein XPO1 in SCLC cell lines and clinical settings.

### 2.5 Elevated HDAC7 Expression Exhibits a Stronger Capacity to Promote β‐catenin Deacetylation and its Nuclear Translocation

**Figure 5:**
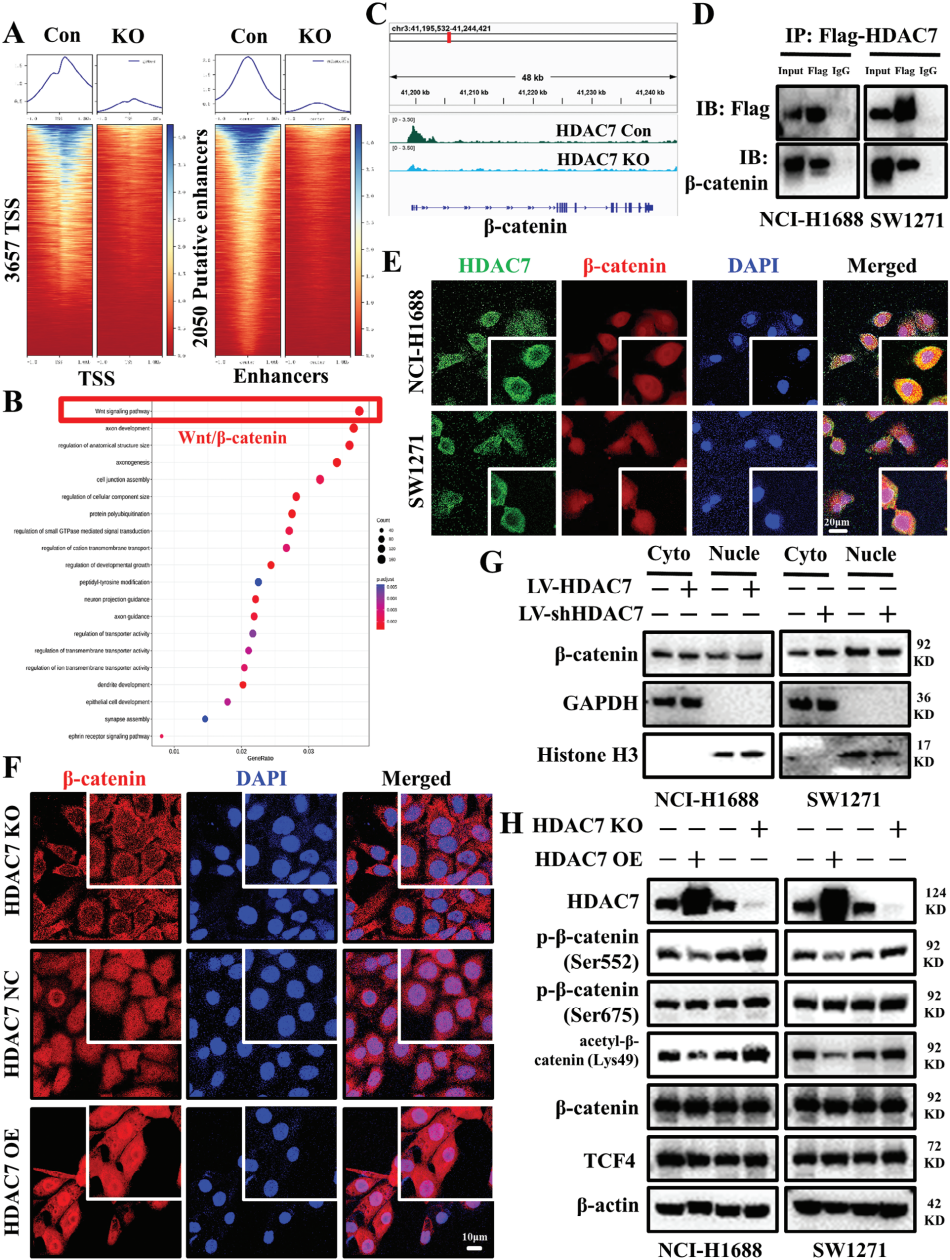
Elevated HDAC7 expression exhibits a stronger capacity to promote β‐catenin deacetylation and its nuclear translocation. A) Heatmap of genomewide H3K27ac cut‐tag analysis revealed enrichment of H3K27ac around TSS (±1 kb) of 3657 genes that was reduced in HDAC7 KO cells. Coverage profiles for H3K27ac enrichment at non‐TSS sites (>1 kb) also showed broad distribution of H3K27ac at 2050 putative enhancers, which was reduced in HDAC7 KO cells. B) GO analysis showed genes associated with peak locations, the Wnt signaling pathway was found to be significantly enriched. C) Cut‐tag profiling in HDAC7 KO cells revealed reduction in H3K27ac peaks near TSS of β‐catenin gene. D) Western blotting of Flag co‐immunoprecipitated HDAC7 and β‐catenin proteins. E) Multicolor immunofluorescence confocal analysis showed significant co‐localization of HDAC7 with β‐catenin in NCI‐H1688 and SW1271 cells. Blue, DAPI. Green, HDAC7. and Red, β‐catenin. Scale bar = 20µm. F) Translation of β‐catenin from cytoplasm to nucleus following HDAC7 overexpression. Blue, DAPI. Red, β‐catenin. Scale bar = 10µm. G) Western blotting detection of β‐catenin distribution in the cytoplasm and nucleus after HDAC7 overexpression or KO. H) Western blot analysis of HDAC7 overexpression‐ or KO‐induced changes in phosphorylation of β‐catenin at Ser552 or acetylation of β‐catenin at Lys49.

We further analyzed the genome‐wide changes in H3K27ac of HDAC7 control and KO cell lines of NCI‐H1688. Hence, we carried out cut & tag analysis in these cell lines and found 3657 H3K27ac peaks at TSS and 2050 peaks at putative enhancers that are decreased with HDAC7 KO cell lines (**Figure** **5**A). Based on the GO analysis of genes associated with peak locations, the Wnt signaling pathway was found to be significantly enriched, suggesting that β‐catenin plays a crucial role in the regulation of SCLC progression by HDAC7. This finding highlights the importance of β‐catenin in mediating the effects of HDAC7 on the progression of SCLC (Figure 5B). In particular, there was a decrease in H3K27ac at the promoter‐proximal regions of *β‐catenin* gene in HDAC7 KO NCI‐H1688 cell line, which was not observed in HDAC7 control cells (Figure 5C). Since loss of β‐catenin resulted in a reduction of primary tumor growth, tumor invasion, and metastasis formation, which is reflected by stalled cell cycle progression.^[^ ^35^ ^]^ In addition, β‐catenin activation has been shown to be a critical transcription factor in HDAC7‐stimulated FGF18 regulation in NSCLC^[^ ^26^ ^]^ and c‐Myc expression in ESCC,^[^ ^25^ ^]^ which are consistent with our findings. We hypothesized that β‐catenin is also a key factor in mediating HDAC7‐dependent cell cycle progression via regulating XPO1 expression and c‐Myc. To determine whether HDAC7 physically interacted with β‐catenin in SCLC, we transfected NCI‐H1688 and SW1271 cells with FLAG‐tagged HDAC7. Co‐immunoprecipitation analysis indicated that HDAC7 bound to β‐catenin (Figure 5D). Our confocal immunofluorescence staining results further confirmed the subcellular colocalization of HDAC7 and β‐catenin in both NCI‐H1688 and SW1271 cells (Figure 5E). We constructed NCI‐H1688 cell lines with stable overexpression and knockout of HDAC7, and subsequently analyzed the subcellular distribution patterns of β‐catenin in these cell lines, along with a control group, using fluorescent immunostaining. Our observations revealed that in the HDAC7 KO group, β‐catenin was predominantly confined to the cytoplasm. Conversely, in the HDAC7 OE group, β‐catenin was found to translocate into the nucleus. These findings suggest that as the expression levels of HDAC7 increase, the subcellular localization of β‐catenin undergoes a gradual shift from the cytoplasm to the nucleus (Figure 5F). Quantitative analysis indicated that ≈72.3% of HDAC7 OE group and ≈52.3% NC group exhibited nuclear localization of β‐catenin, compared with ≈17.6% in HDAC7 KO group (Figure S1D, Supporting Information). The nuclear–cytosolic protein assay confirmed that HDAC7 overexpression promoted β‐catenin nuclear import, while HDAC7 KO inhibited β‐catenin nuclear import partly (Figure 5G,E,F). It is known that several tyrosine residues of human β‐catenin undergo phosphorylation.^[^ ^36^ ^]^ We therefore determined whether HDAC7 KO or OE affected phosphorylation of these tyrosine residues. HDAC7 KO and OE resulted in marked changes in phosphorylation of Ser552; however, it did not affect total β‐catenin protein levels (Figure 5H). Previous studies have reported that β‐catenin deacetylation (Lys49) inhibits its own phosphorylation, thus, activating β‐catenin.^[^ ^25^, ^37^ ^]^ We found that overexpression of HDAC7 downregulated β‐catenin acetylation (Lys49) and phosphorylation (Ser552), while HDAC7 KO upregulated β‐catenin acetylation (Lys49) and phosphorylation (Ser552) (Figure 5H). These results demonstrate that HDAC7 not only directly interacts with β‐catenin to mediate its deacetylation, but furthermore, increased expression of HDAC7 exhibits an enhanced ability to facilitate both the deacetylation and nuclear translocation of β‐catenin.

### 2.6 The Intricate Interplay between HDAC7, c‐Myc, and XPO1

**Figure 6:**
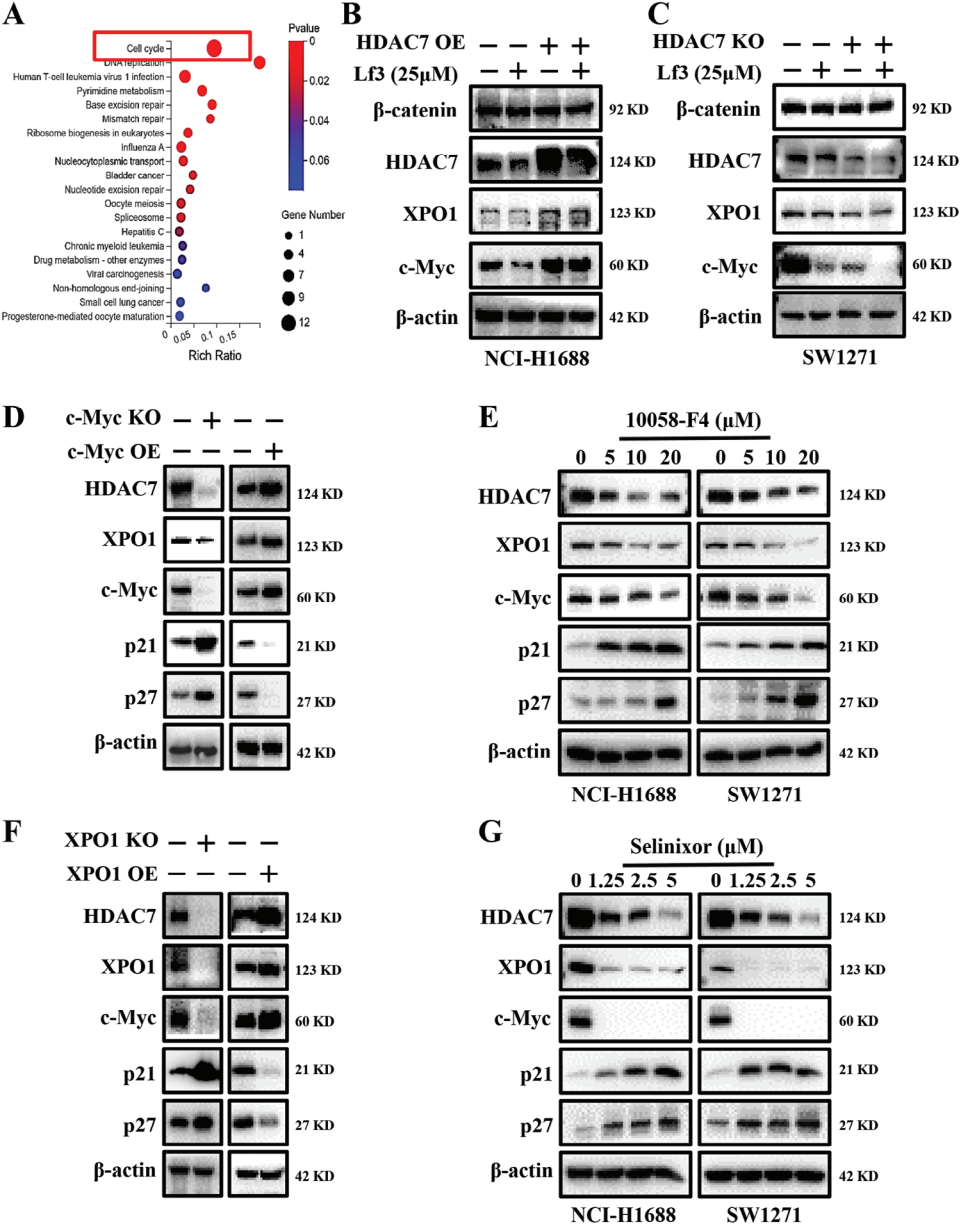
The intricate interplay between HDAC7, c‐Myc, and XPO1. A) Signal pathway enrichment analysis of RNA‐sequencing in HDAC7 control and KO NCI‐H1688 cells. B) Effect of β‐catenin inhibitor Lf3 (25 µM) on HDAC7 overexpression‐induced HDAC7, XPO1, and c‐Myc upregulation. C) Enhancement of β‐catenin inhibitor Lf3 (25 µM) on HDAC7 KO‐induced HDAC7, XPO1, and c‐Myc downregulation. D) Changes in HDAC7, XPO1, c‐Myc, p21, and p27 protein levels following c‐Myc KO or overexpression. E) Effect of c‐Myc inhibitor 10058‐F4 on HDAC7, XPO1, c‐Myc, p21, and p27 protein levels. F) Changes in HDAC7, XPO1, c‐Myc, p21, and p27 protein levels following XPO1 KO or overexpression. G) Effect of XPO1 inhibitor selinexor on HDAC7, XPO1, c‐Myc, p21, and p27 protein levels.

We further analyzed mRNA sequencing profiles of HDAC7 control and KO cell lines of NCI‐H1688. Bubble plots showed that the cell cycle was the top‐ranked pathway (**Figure** **6**A). In addition, we treated HDAC7 overexpression and control cells with Lf3; a robust specific antagonist of the β‐catenin/TCF4 interaction. Lf3‐treatment (25 µM) not only reduced the upregulation of downstream proteins XPO1 and c‐Myc induced by basal or overexpressed levels of HDAC7, but interestingly, we also found that Lf3 reduced the expression level of HDAC7, an upstream protein of β‐catenin, in both HDAC7‐overexpressing and control cell lines (Figure 6B,C), suggesting maybe there is a close relationship among these three proteins. To verify the relationship among HDAC7, c‐Myc, and XPO1, we generated c‐Myc KO, c‐Myc overexpression (OE), XPO1 KO, and XPO1 OE NCI‐H1688 cell lines. Western blotting showed that c‐Myc KO resulted in a reduction of HDAC7 protein levels, which was accompanied by a decrease in XPO1. Additionally, c‐Myc OE positively correlated with both upregulation of HDAC7 and XPO1 (Figure 6D). For further conformation, we treated NCI‐H1688 and SW1271 cells with 10058‐F4, a c‐Myc specific inhibitor, which led to significant reduction of HDAC7 and XPO1 (Figure 6E). To determine the effect of XPO1 on expression of HDAC7 and c‐Myc, we used XPO1 KO, XPO1 OE, and their control cells. Western blotting showed that XPO1 KO resulted in reduction of HDAC7 protein, which was accompanied by a decrease in c‐Myc. XPO1 OE positively correlated with upregulation of HDAC7 and c‐Myc (Figure 6F). To confirm this this, we treated NCI‐H1688 and SW1271 cells with selinexor (KPT330), a selective XPO1 inhibitor. Western blotting indicated that XPO1 inhibition led to marked decline of HDAC7 and c‐Myc proteins (Figure 6G). To validate the significance of HDAC7/c‐Myc/XPO1 signaling in regulating SCLC proliferation, we investigated the consequences of individual and combined knockouts of c‐Myc, XPO1, and c‐Myc & XPO1 in NCI‐H1688 cells that stably overexpress HDAC7. After knocking out HDAC7, c‐Myc, and XPO1, we observed a partial reversal of the enhanced cell growth induced by HDAC7 overexpression (Figure S1G,H, Supporting Information). Additionally, there was a further reduction in the levels of c‐Myc, XPO1, and ectopic HDAC7 proteins in the HDAC7‐overexpressing NCI‐H1688 cell lines (Figure S1I, Supporting Information). Notably, compared to the HDAC7‐overexpressing (OE) cells treated with selinexor, the c‐Myc knockout (KO), XPO1 KO, and simultaneous c‐Myc & XPO1 KO groups significantly potentiated the antiproliferative effect of selinexor against HDAC7 OE NCI‐H1688 cells (Figure S1G,H, Supporting Information). Furthermore, these KO groups inhibited the expression of HDAC7, c‐Myc, and XPO1 proteins (Figure S1I, Supporting Information). These findings underscore the mutual influence of HDAC7, c‐Myc, and XPO1 expression levels and highlight that targeting the HDAC7/c‐Myc/XPO1 axis impedes SCLC cell growth.

### 2.7 Both XPO1 and c‐Myc Serve as Target Genes of β‐catenin, and Notably, XPO1 is additionally a Target Gene of c‐Myc

**Figure 7:**
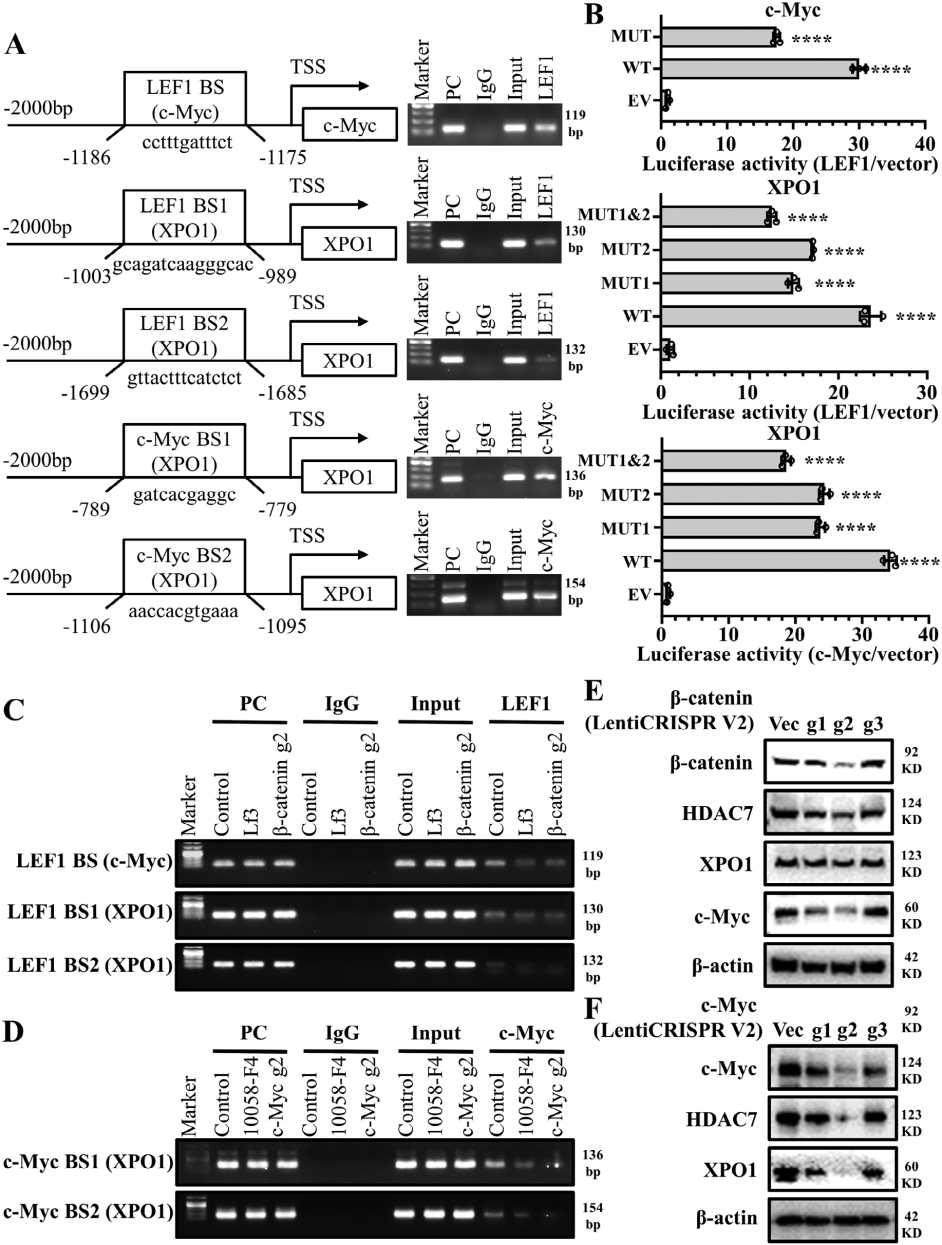
Both XPO1 and c‐Myc serve as target genes of β‐catenin, and notably, XPO1 is additionally a target gene of c‐Myc. A) Ch‐IP assay was performed to validate binding of LEF1 and c‐Myc to promoter of indicated genes in NCI‐H1688 cell line. B) Dual luciferase assay was performed to determine the promoter activity in 293FT cell line. C) Ch‐IP assay was performed to validate changes in binding ability of LEF1 to promoter of c‐Myc and XPO1 under Lf3 treatment or β‐catenin KO in NCI‐H1688 cell line. D) Ch‐IP assay was performed to validate changes in binding affinity of c‐Myc to promoter of XPO1 under 10058‐F4 treatment or c‐Myc KO in NCI‐H1688 cell line. E) Western blotting analysis showed changes in the expression of HDAC7, XPO1, and c‐Myc after β‐catenin KO. F) Western blotting analysis illustrating expression of HDAC7, XPO1, and c‐Myc after c‐Myc KO.

Previous studies reported that c‐Myc is the downstream target of β‐catenin, but the DNA‐binding site of c‐Myc promoter is not clear, less is known about which genes or transcription factors regulate XPO1. We found a potential lymphoid enhancer‐binding factor (LEF)1 binding site in the promoter of c‐Myc [LEF1 BS (c‐Myc)]; two potential LEF1 binding sites in the promoter of XPO1 [LEF1 BS1 (XPO1) and LEF1 BS2 (XPO1)], and two potential c‐Myc binding sites of XPO1 in promoters of XPO1 [c‐Myc BS1 (XPO1) and c‐Myc BS2 (XPO1)] via the online transcription factor prediction software, JASPAR (**Figure** **7**A, left). Chromatin immunoprecipitation (Ch‐IP) assay showed that LEF1 strongly bound to the predicted site of the c‐Myc promoter, as well as the predicted sites of the XPO1 promoter. c‐Myc bound to the two predicted binding sites of XPO1 gene promoter regions (Figure 7A, right). Luciferase assays with constructs harboring mutations in each transcription factor binding site confirmed that each site was needed for full promoter activity (Figure 7B). To further confirm our new findings, we constructed β‐catenin stable KO cell lines or treated wild‐type cells with β‐catenin‐specific inhibitor (Lf3). Binding affinity of LEF1 transcription factor to potential binding sites in the promoter regions of target genes was reduced (Figure 7C), and XPO1 and c‐Myc protein expression was downregulated (Figures 6B,C, and 7E). We constructed stable c‐Myc KO NCI‐H1688 cells or treated NCI‐H1688 cells with c‐Myc specific inhibitor 10058‐F4. Similarly, we found that the binding affinity of c‐Myc transcription factors to potential binding sites in the XPO1 promoter region was inhibited (Figure 7D), and XPO1 protein level was significantly suppressed (Figures 6D,F, and 7F). These results suggest that β‐catenin can promote c‐Myc and XPO1 expression by binding to the promoter regions of c‐Myc and XPO1, while c‐Myc, as a transcription factor, can bind to the promoter region of XPO1 to promote XPO1 expression.

### 2.8 A Highly Specific XPO1 Inhibitor Demonstrates Superior Antitumor Efficacy Compared to a Multi‐Targeted HDAC Inhibitor both In Vivo and In Vitro

**Figure 8:**
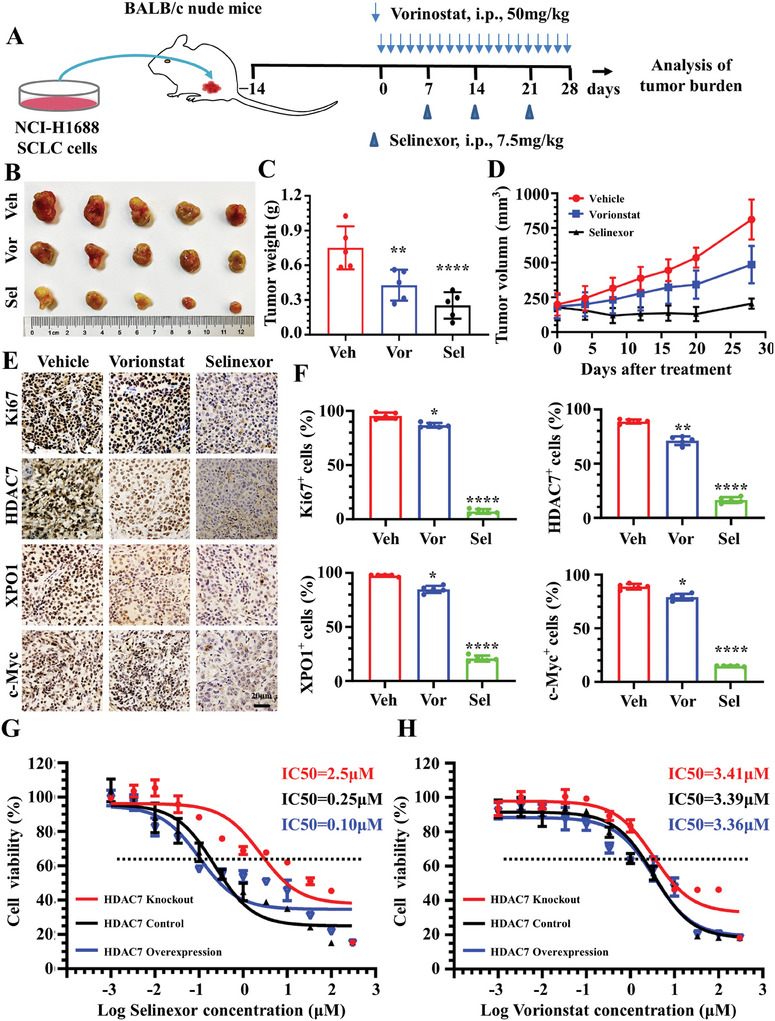
A highly specific XPO1 inhibitor demonstrates superior antitumor efficacy compared to a multi‐targeted HDAC inhibitor both in vivo and in vitro. A) Schematic diagram of animal experiments. B) Typical images of tumors treated with vehicle, vorinostat, and selinexor; Ruler unit: cm. C) Box plots of tumor tissue weights in vehicle‐, vorinostat‐, and selinexor‐treated mice (*n* = 5 per group). D) Tumor volume analysis of mice in vehicle, Vorinostat, and selinexor groups. E) Typical images of Ki67, HDAC7, XPO1, and c‐Myc immunostaining of cross‐sections of tumors of vehicle‐, vorinostat‐, and selinexor‐treated mice; Brown, Ki67, HDAC7, XPO1 and c‐Myc; Blue, nuclei; Scale bar, 20 µm. F) Quantitative analysis of the ratio of Ki67‐, HDAC7‐, XPO1‐, and c‐Myc‐positive cells in cross‐sections of tumors. G) IC50 analysis of selinexor sensitivity by CCK‐8 assay in HDAC7 control, HDAC7 KO, and HDAC7 OE stable cell lines of NCI‐H1688. H) Dose–response curves determined by CCK‐8 assay were used to calculate the IC50 of vorinostat in HDAC7 control, HDAC7 KO, and HDAC7 OE stable cell lines of NCI‐H1688.

Our results suggest that the HDAC7/c‐Myc/XPO1 axis is a potential therapeutic target for SCLC. First, given that c‐Myc has been considered undruggable to date. Second, vorinostat is a potent pan‐inhibitor of HDAC1, HDAC2, and HDAC3 (Class I), HDAC6 and HDAC7 (Class II), and HDAC11 (Class IV), and there has been no clinically available selective inhibitors of HDAC7 until now. Third, the only accessible single‐target drug in this axis is selinexor against XPO1. Nonetheless, using these two clinically accessible drugs, we compared the antitumor effects of selinexor and vorinostat in HDAC7‐positive NCI‐H1688 cells (**Figure** **8**A). Selinexor (7.5 mg kg^−1^) yielded a significant reduction in tumor burden compared with vorinostat (50 mg kg^−1^) (Figure 8B,–D). To determine the effect of different treatments on HDAC7, c‐Myc, XPO1, and ki67, we performed IHC staining of these proteins in each group and found that they were markedly reduced in the selinexor group compared to the vorinostat (Figure 8E). Quantitative analysis indicated that positive ratio of these markers in different groups (Figure 8F). The half‐maximal inhibitor concentration (*IC50*) was dramatically decreased in HDAC7 OE (0.1 µM) and HDAC7 control (0.25 µM) cell lines, compared with HDAC7 KO NCI‐H1688 (2.5 µM) cells. Further analysis revealed a positive correlation between the sensitivity of SCLC cell lines to selinexor and expression of HDAC7 (Figure 8G). In contrast to the response to selinexor of SCLC cells with different HDAC7 expression levels, we did not find a correlation between the level of HDAC7 and vorinostat (Figure 8H). This may have been because vorinostat is a multi‐target inhibitor of HDACs, which with low IC50 values of 10 nM and 20 nM for HDAC1 and HDAC3, respectively, but with high IC50 valves for HDAC7. These results suggest that XPO1 inhibition induced by selinexor shows more significant inhibition in HDAC7‐overexpressing tumors compared with vorinostat. In addition, SCLC cell lines with high HDAC7 levels were more sensitive to selinexor, but not vorinostat, suggesting that HDAC7 could serve as a marker for screening populations that could benefit from selinexor treatment.

### 2.9 Overexpression of HDAC7 Enhances the Sensitivity of Organoids Derived from Different Cancer Types to Selinexor Treatment

**Figure 9:**
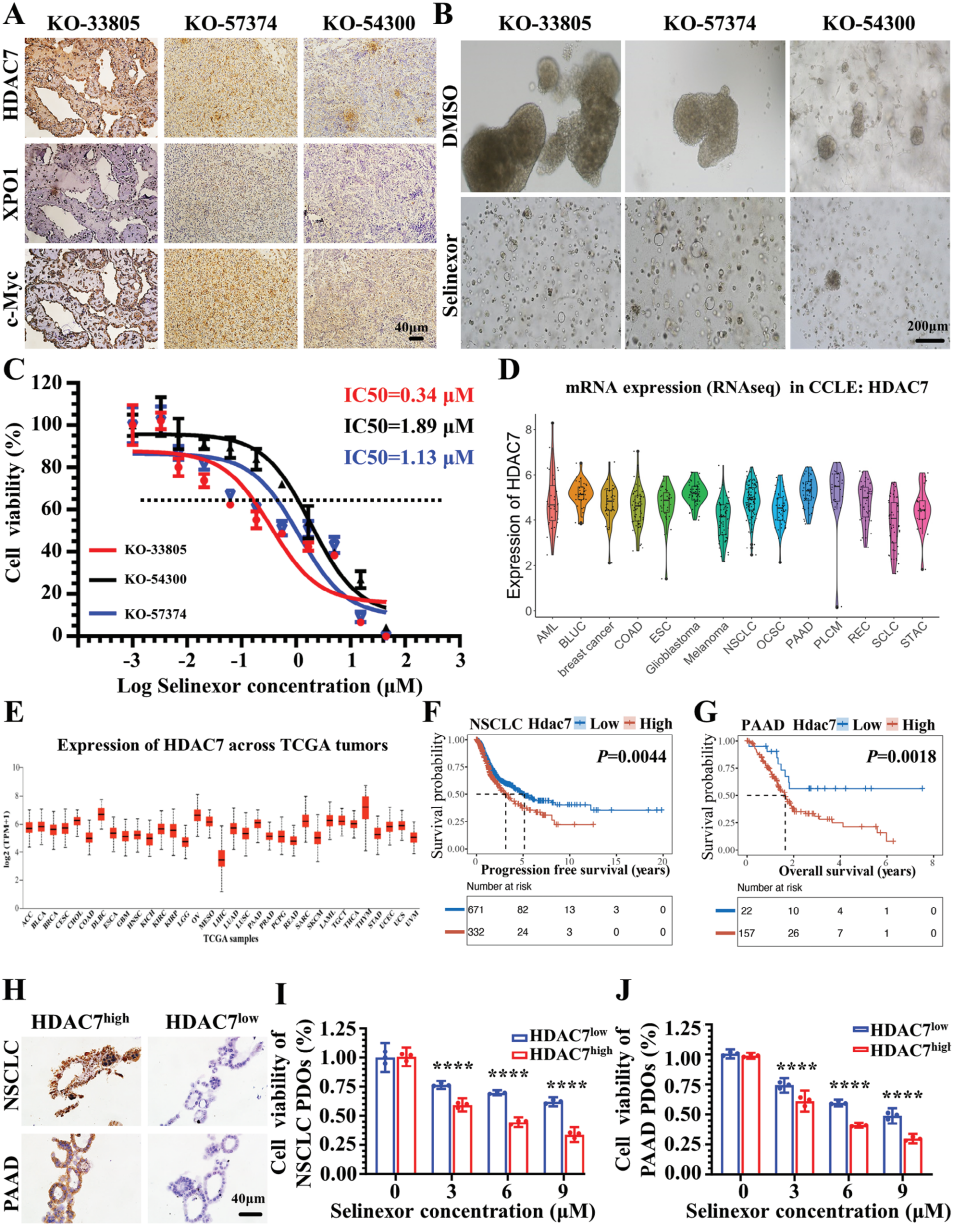
Overexpression of HDAC7 enhances the sensitivity of organoids derived from different cancer types to selinexor treatment. A) HDAC7, XPO1, and c‐Myc immunostaining of PDOs from SCLC patients; Brown, HDAC7, XPO1 and c‐Myc; Blue, nuclei; Scale bar, 40 µm; B) Representative bright‐field images of PDOs treated with selinexor; Scale bar, 200 µm. C) Dose–response curves determined by the CellTiter‐Glo3D Cell Viability assays were used to calculate the IC50 of selinexor in KO‐33805, KO‐57374, and KO‐54300 PDOs. D) HDAC7 mRNA expression in different cancer cell lines. Data from the CCLE database (https://sites.broadinstitute.org/ccle/). E) HDAC7 mRNA expression in different cancer types. Data from the UALCAN database (https://ualcan.path.uab.edu/index.html). F,G) Kaplan–Meier analysis of HDAC7 expression with OS in NSCLC cohort (F) and PAAD cohort (G) of the TCGA. The cutoff value of HDAC7 expression was based on the minimum *P*‐value approach. H) Representative images of NSCLC and PAAD PDOs with positive or negative HDAC7 expression. Brown, HDAC7; Blue, nuclei; Scale bar, 40 µm. I) Absolute fold changes in NSCLC PDOs with positive or negative HDAC7 expression treated with selinexor. J) Absolute fold changes in PAAD PDOs with positive or negative HDAC7 expression under selinexor treatment.

To investigate the efficacy of selinexor in SCLC patients with HDAC7 overexpression, we used PDOs from SCLC patients with HDAC7 upregulation (KO‐33805), and SCLC patients with low expression of HDAC7 (KO‐57374 and KO‐54300) (**Figure** **9**A). Immunohistochemical staining of these PDOs showed a positive correlation between HDAC7, XPO1, and c‐Myc expression, which is consistent with what we found in clinically stained specimens. The PDO with HDAC7 overexpression (KO‐33805) was significantly more sensitive to selinexor than the corresponding PDOs with low HDAC7 expression (KO‐57374 and KO‐54300) (Figure 9B). The IC50 of selinexor was 0.34 µM in KO‐33805 (Figure 9C), and 1.13 and 1.89 µM in KO‐57374 and KO‐54300, respectively (Figure 9C). Compared with KO‐57374 and KO‐54300, KO‐33805 was markedly more sensitive to selinexor (Figure 9C). These results suggest that SCLC with high HDAC7 expression has better responsiveness to selinexor. NSCLC and pancreatic adenocarcinoma (PAAD) also had high expression of HDAC7 in the CCLE data (Figure 9D) and TCGA (Figure 9E). We found that NSCLC patients with high HDAC7 expression exhibited poorer survival probability compared with patients with low HDAC7 expression (*p* = 0.0044) (Figure 9F). A similar result was observed in PAAD patients (*p* = 0.018) (Figure 9G). We examined the correlations between HDAC7 expression and responsiveness to selinexor in NSCLC and PAAD PDOs. IHC revealed one positive and one negative organoid with HDAC7 expression derived from patients with NSCLC or PAAD (Figure 9H). NSCLC and PAAD PDOs with high HDAC7 expression were more sensitive to selinexor compared with low HDAC7 expression (Figure 9I,J). These results suggest that HDAC7 plays a critical role in sensitivity of selinexor in patients with SCLC, NSCLC, or PAAD.

## 3 Discussion

We have identified a novel SCLC subtype, characterized by pronounced HDAC7 overexpression, which is consistently observed across different SCLC subtypes, with the highest prevalence in the SCLC‐I subtype. Our findings reveal that HDAC7 upregulates the expression of XPO1 and c‐Myc in SCLC cells, thereby promoting cell proliferation, colony formation, and tumor progression. Mechanistically, HDAC7 deacetylates β‐catenin at lysine 49, modulating its phosphorylation at Ser552, and facilitating β‐catenin's nuclear translocation. In the nucleus, β‐catenin forms complexes with TCF/LEF1 transcription factors, which subsequently bind to the promoter regions of XPO1 and c‐Myc, enhancing their expression. Furthermore, c‐Myc directly binds to the XPO1 promoter, further augmenting XPO1 expression. Collectively, these findings suggest an HDAC7/XPO1/c‐Myc positive feedback loop that drives SCLC proliferation and tumor progression. Importantly, we demonstrate for the first time that HDAC7, as a promising biomarker for selinexor sensitivity, has the potential to identify SCLC patients who may benefit from selinexor therapy.

SCLC is an aggressive neuroendocrine tumor with a poor OS rate, which has proven a challenge in the era of personalized therapy, due partly to underappreciated inter‐ and intratumoral heterogeneity.^[^ ^6b,c^ ^]^ Abnormal expression of HDAC7 has been shown in various cancers and is involved in pathophysiological processes such as tumor cell proliferation, migration and apoptosis.^[^ ^24^, ^25^, ^26^, ^28^ ^]^ Moreover, HDAC7 expression level has a significant negative correlation with patient prognosis in ESCC^[^ ^25^ ^]^ and NSCLC.^[^ ^26^ ^]^ However, the prognostic role and function of HDAC7 in SCLC are still unclear. Our study showed that elevated HDAC7 expression in SCLC was strongly correlated with the SCLC‐I subgroup and facilitated disease progression. Notably, we also found HDAC7 overexpression in SCLC‐P and SCLC‐A subgroups. Survival analyses indicated that high expression of HDAC7 was an independent negative prognostic factor for SCLC patients. Vorinostat, a pan‐HDAC inhibitor targeting HDAC7 and other HDACs, has been used for the treatment of refractory cutaneous T‐cell lymphoma^[^ ^38^ ^]^ and acute myeloid leukemia.^[^ ^39^ ^]^ Therefore, HDAC7 has the potential to serve as a prognostic indicator and target for SCLC treatment.

In this study, we showed that HDAC7 promoted SCLC progression. Recent studies have revealed that oncogenic actions of HDAC7 are dependent on the c‐Myc amplification,^[^ ^25^, ^29^, ^40^ ^]^ because c‐Myc helps tumor cells escape from cellular senescence process and promotes tumor cell growth via inhibiting p21/p27 expression, thus accelerating G1–S cell cycle transition.^[^ ^25^, ^29^ ^]^ Looking for genes downstream of HDAC7 in SCLC, *c‐Myc*, *MMP2*, *DPT*, *C7*, *C3*, *CCL21*, and *PTGDS*, were selected as the candidates. Among these genes, *c‐Myc* had the most significant change and the smallest *p* value when considering the George et al. cohort^[^ ^33^ ^]^ and GSE60052.^[^ ^34^ ^]^ Our further analysis confirmed that c‐Myc was directly upregulated by HDAC7 in SCLC. To investigate further the molecular effects of HDAC7, we conducted gene expression analysis by RNA‐sequencing, gene set enrichment analysis showed significant enrichment of MYC target genes, which included transcripts associated with XPO1 functions such as c‐Myc RNA translocation from nucleus to cytoplasm. XPO1, also known as CRM1, is an important transporter of more than 220 cargo proteins^[^ ^41^ ^]^ and a wide range of mRNAs (over 3000).^[^ ^42^ ^]^ Our previous study identified and validated XPO1 as a putative new therapeutic target for SCLC, and further exploration revealed that XPO1 inhibition synergizes with PARP1 inhibition in SCLC by targeting nuclear transport of forkhead box O3a.^[^ ^9^ ^]^ Interestingly, we discovered that not only the β‐catenin inhibitor Lf3 could inhibit the expression of HDAC7, XPO1, and c‐Myc, but also the c‐Myc specific inhibitor 10058‐F4 and the XPO1 specific inhibitor selinexor suppressed the expression of these proteins. Downregulation of expression levels of the three proteins in the HDAC7/XPO1/c‐Myc axis was achieved by different target inhibitors. Thus, specific inhibitors against these three different targets may offer an interesting approach to inhibit SCLC tumor progression. Our findings are of clinical significance in developing new interventions for SCLC.

The activation status of the β‐catenin signaling pathway in SCLC promotes tumor proliferation and colony formation. In this study, we showed that HDAC7 bound to β‐catenin directly and deacetylated β‐catenin at Lys49, which is consistent with previous studies.^[^ ^25^, ^26^ ^]^ Previous studies have demonstrated that β‐catenin Lys49 deacetylation leads to β‐catenin dephosphorylation at Ser675 in ESCC and NSCLC^[^ ^25^, ^26^ ^]^ or Ser45 in glioma cells.^[^ ^43^ ^]^ We found that HDAC7 overexpression hindered β‐catenin phosphorylation at Ser552 in SCLC cells. In this study, we showed that HDAC7 mediates c‐Myc and XPO1 via stimulating β‐catenin signals and forming complexes with TCF/LEF1 transcription factions. Another finding was that upon stimulation by HDAC7, β‐catenin bound to the promoter regions of c‐Myc and XPO1, leading to the upregulation of c‐Myc and XPO1 expression. Meanwhile, c‐Myc directly bound to the promoter region of XPO1 to promote XPO1 expression.

Organoids formed from cancer patients can recapitulate patient responses in the clinic and are now used for evaluating drug sensitivity in various types of cancer, including SCLC.^[^ ^44^ ^]^ Thus, we confirmed the relationship between the HDAC7/XPO1/c‐Myc ais and selinexor sensitivity in three SCLC PDO models (KO‐57374, KO‐54300 and KO‐75345). Similar to the SCLC cell lines, we observed that the PDO with high HDAC7 expression was more sensitive to selinexor than the other PDOs with low HDAC7 expression. To conform the clinical significance of HDAC7, we treated NSCLC and PAAD PDOs with selinexor, and found that selinexor showed greater inhibition in PDOs with high expression of HDAC7. To our knowledge, this study is the first to propose HDAC7 expression levels to determine innate sensitivity to selinexor in SCLC, NSCLC, and PAAD PDOs.

There are certain limitations to our research. First, our findings have only been validated in SCLC tumor‐bearing mice and PDOs, but not in PDX (patient‐derived xenograft) models. This restricts the generalization of our results to a broader patient population. Second, since our current results have yet to undergo clinical validation, we are embarking on an innovative clinical umbrella study, pioneering the exploration of molecular subtypes in SCLC, with a dedicated focus on the HDAC7‐positive SCLC subtype, as part of our subsequent follow‐up research. This endeavor will provide crucial insights into the clinical applicability and relevance of our discoveries.

## 4 Conclusion

In summary, HDAC7‐high SCLC subtype is linked to proliferation and poor prognosis, and high HDAC7 level increases sensitivity to selinexor both in vitro and PDOs. Mechanistically, our results indicate that HDAC7 can promote SCLC tumorigenesis by stimulating XPO1 and c‐Myc gene expression. This activity is mediated by β‐catenin translocation and subsequent formation of TCF/LEF1 transcription complexes that bind to promoters of target genes. We also discovered that the XPO1 promoter region contained binding sites for the transcription factor c‐Myc in addition to TCF/LEF1, and further experiments confirmed that c‐Myc upregulated expression of XPO1. Considering the accessibility and specificity of clinical drugs, we chose XPO1 inhibitor selinexor, and found that HDAC7 can be used as a marker to screen patients who may benefit from selinexor treatment. Based on these results, we propose selinexor as a therapeutic option in SCLC patients with high HDAC7 expression.

## 5 Experimental Section

### Study Design

The aim of this study was to identify novel features of HDACs involved in SCLC progression as well as define the mechanism of sensitivity to XPO1 inhibition in the HDAC7‐positive subtype of SCLC. This objective was accomplished by i) uncovering the correlation between SCLC subtypes and HDAC7 expression, ii) validating of the relationship between HDAC7 level and proliferation and progression in vitro and in vivo, iii) dissecting the mechanism by which HDAC7 controls XPO1 and c‐Myc, verifying the interplay among these three proteins, and thereby gaining insights into their influence on the cell cycle in SCLC, iv) determining the role of HDAC7 levels in reflecting sensitivity to the XPO1 inhibitor selinexor, and v) conducting a series of in vitro and PDOs studies to delineate the therapeutic potential of selinexor in HDAC7‐high SCLC subtype, recognizing that high HDAC7 levels augment sensitivity to selinexor.

### Patients and Tissues Samples

A total of 110 SCLC tumor tissues were from patients who had undergone complete surgical resection after histological diagnosis of SCLC at Tianjin Medical University Cancer Institute and Hospital, China. This study was approved by the institutional Ethics Committee to use these specimens and patient data. All patients provided written consent for the use of their specimens and data.

### Reagents

SAHA (HY‐18361), Lf3 (HY‐101486), and 10058‐F4 (HY‐12702) were purchased from MedChemExpress. Selinexor (S7252) was from Selleck. HDAC7, XPO1, p21, p27, Flag, β‐catenin, p‐β‐catenin, acetyl‐β‐catenin, histone H3, Ki67, and β‐actin were purchased from Cell Signaling Technology (Danvers, MA, USA). c‐Myc, GAPDH, and TCF4 were purchased from Proteintech (Chicago, IL, USA). HDAC7 was purchased from Beyotime (Shanghai, China). Horseradish‐peroxidase‐conjugated secondary antibodies were from Cell Signaling Technology. Alexa‐Fluor‐conjugated secondary antibodies were from Invitrogen (Carlsbad, CA, USA). Matrigel was from BD Biosciences (San Diego, CA, USA).

### IHC and Immunofluorescence

IHC was used to examine the expression of HDAC7, XPO1, c‐Myc, and Ki67 in SCLC tissues. The antigens were extracted from formalin‐fixed, paraffin‐embedded tissue specimens by deparaffinization in xylene and heating in a pressure cooker for 2.5 min. The slides were incubated with primary antibodies at 4 °C overnight, followed by secondary antibodies at room temperature for 30 min. Chromogenic reactions were performed using a DAB kit (ZL1‐9019; ZSGB‐BIO, China). The expression of HDAC7, XPO1, c‐Myc, and Ki67 was evaluated using an H‐score system. The H‐score, ranging from 0 to 300, was calculated based on the ratio of the weighted sum of positive cells to the total number of detected cells, taking into account the staining intensity. The staining intensity was assessed using the following criteria: 0 for negative, 1 for weak positive, 2 for moderate positive, and 3 for strong positive. Three independent clinical pathologists, blinded to all patient information, evaluated all IHC results. Additionally, immunofluorescence staining was conducted on SCLC cells, where the cells were plated on coverslips and incubated overnight at 4 °C with an anti‐β‐catenin antibody. Then, the cells were incubated with fluorescently labeled secondary antibody at room temperature for 1 h. The coverslips were then sealed by SlowFade Gold Antifade Mountant (Invitrogen). Images were captured with a confocal fluorescence microscope.

### Culture and Lentivirus Infection of Human SCLC Cell Lines

Human SCLC cell lines (NCI‐H1688, DMS53, SHP77, NCI‐H889, NCI‐H209, NCI‐H524, NCI‐H446, DMS114, SW1271, and NCI‐H196) were purchased from American Type Culture Collection (Manassas, VA, USA). All cells were cultured in suggested medium (RPMI1640 or DMEM) (Gibco, USA) supplemented with 10% fetal bovine serum, and 100 µg mL^−1^ streptomycin and penicillin. Lentivirus infection of NCI‐H1688 and SW1271 cells was conducted according to the protocol. For the cell lines with overexpressed targeted genes, HDAC7, XPO1, and c‐Myc genes were cloned into pLenti‐CMV‐RFP‐BSD vectors, respectively. Lentiviruses were cultivated in 293T cells for stable transfection of the cell lines, and an empty vector was transfected into cells as a control. A total of 1 × 10^5^ tumor cells in 2 mL medium with 8 µg mL^−1^ polybrene were infected with 1 mL lentivirus supernatant. After 48 h, blasticidin (4 µg mL^−1^) was added for selection. For the cell lines with stable knockout, sgRNA sequences (Table S1, Supporting Information) targeting HDAC7, β‐catenin, XPO1, and c‐Myc were obtained from the sgRNA design tool (http://sam.genome‐engineering.org/database/, Cas9‐Activators with SAM) and cloned into lentiCRISPR v2. Target cells were transfected with lentiviral particles followed by puromycin selection (1 µg mL^−1^). Of these three stable cell lines, the most efficient one was used for the relevant assays.

### In Vivo SCLC Tumor Xenograft Assays

6‐week‐old female BALB/c nude mice were purchased from Gempharmatech (Nanjing, China). Experiment (1): NCI‐H1688 HDAC7^Control^ cells [5 × 10^6^ per mouse in 100 µl PBS (1:1 mixed with Matrigel)] or NCI‐H1688 HDAC7^KO^ cells [(5 × 10^6^ per mouse in 100 µl PBS (1:1 mixed with Matrigel)] were separately subcutaneously inoculated into the right/left lower back of the mice (n = 6). Tumor sizes was measured with a dial caliper in a blinded manner every 3 days. Tumor volume = 1/2 (Length × Width^2^). All mice were killed after 25 days, and tumors were excised and weighed for further analysis. Experiment (2): NCI‐H1688 cells [5 × 10^6^ per mouse in 100 µl PBS (1:1 mixed with Matrigel)] were inoculated in the same space as described above. Mice Tumor bearing mice were separated into three groups (n = 5), and treated with vehicle or vorinostat alone (50 mg kg^−1^, i.p., daily, 5 days a week), or selinexor alone (7.5mg kg^−1^, i.p., once a week). Tumor sizes was measured with a dial caliper in a blinded manner. Tumor volume = 1/2 (Length × Width^2^). After 28‐days’ treatment, tumors were harvested for further analysis. These animal experiments were conducted with approved from the Animal Care and Use Committee of Tianjin Cancer Institute and Hospital of Tianjin Medical University.

### Patient‐Derived Organoids Study

PDOs were maintained in a 3D culture system with Matrigel and organoid growth medium (Cat# K2O‐M‐LU) and seeded in 96‐well plates, which were then treated with selinexor at various concentrations for 5 days. Images were taken by Leica DMil. Cell viability was determined using the CellTiter‐Glo3D Cell Viability Assay (Promega). Dose‐effect curves were generated via GraphPad Prism software (version 9.5). PDOs provided by K2 Oncology Inc, (Beijing, China).

### Colony Formation Assay

A total of 1000 NCI‐H1688 or SW1271 cells were seeded in six‐well plates for 14 days. Colonies were fixed with formalin (Solarbio, Beijing, China) for 10 min and stained with 0.1% crystal violet (Salarbio, Beijing, China). The colonies containing >50 cells were counted for further analysis.

### EdU Incorporation Assay

The BeyoClick Edu Cell kit with Alexa Fluor 555 (Beyotime, Shanghai, China) was used to detect the newly synthesized DNA and evaluate cell proliferation. Images were obtained using an Olympus FV1000 confocal microscope (Olympus, Tokyo, Japan). EdU‐positive cells were manually counted and expressed as percentages of all cells, which were stained by Hoechst 33 342.

### Western Blotting Analysis

SCLC cells were homogenized and subjected to SDS‐PAGE. The proteins were transferred onto a polyvinylidene difluoride membrane (Roche Molecular Biochemicals, Quebec, Canada), blocked with 5% milk powder in TBS‐T buffer (20 mM Tris‐HCl, pH 7.4, 137 mM NaCl, and 0.1% Tween) for 1 h at room temperature, incubated overnight at 4 °C with appropriate primary antibodies against the target proteins, further incubated with horseradish‐peroxidase‐conjugated secondary antibodies, and developed with the ECL System (Millipore, Billerica, MA, USA).

### RNA Sequencing

Total RNA were extracted from NCI‐H1688 HDAC7^Control^ or NCI‐H1688 HDAC7^KO^ cells using Trizol reagent (QIAGEN, USA). All samples were sent to BGI (Wuhan, China) for further RNA sequencing and analysis via Illumina HiSeq 2500 (San Francisco, CA, USA). The expression level of each gene was calculated as the fragments per kilobase of transcript per million mapped reads (FPKM) value. Differentially expressed genes (DEGs) between groups were identified by the edgeR package (http:// www. rproject.org/) with thresholds of false discovery rate <0.05 and absolute log2‐fold change ≥1.

### CUT&Tag Analysis

The CUT&Tag experiments were performed by Wuhan Zhenyue Bioinformatics Co., Ltd. Quality control and trimming of the reads were done using FastQC (v0.11.9) Clean reads were aligned to the human genome (hg38) using Bowtie2(v2.4.5). PCR duplicates were removed using Picard MarkDuplicates. Peak calling was performed using Macs2. Peaks were filtered at a q‐value threshold < 0.05. Peak calls from each replicate across both controls and treatments were merged into a union set using bedtools merge. Differential peaks were identified using DESeq2(v1.32.0) with default parameters. Regions with a *P*‐value threshold < 0.05(Log2FoldChange > 0.5 or < −0.5) were considered to be statistically significant. Heatmaps were produced using ComplexHeatmap. Peaks were annotated to the nearest feature using ChIPseeker (v1.32.0). GO analysis was performed using clusterProfiler (v4.0.0).

### Immunoprecipitation Analysis

Immunoprecipitation assay was performed using the Pierce Classic Magnetic IP/Co‐IP Kit (Thermo Scientific Pierce) according to manufacturer's instruction. Briefly, 1 mg of SCLC cells determined by the Pierce BCA protein assay. Then the supernatants were incubated with of Flag (10 µg) antibody (Cell Signaling Technology) and 25 µL of Pierce protein A/G Magnetic beads overnight at 4 °C with gentle rotation. The beads were rinsed twice with lysis buffer and ultrapure water once, boiled in 1× Lane Marker Sample Buffer, and subjected to SDS‐PAGE and western blotting.

### Quantitative Real‐Time PCR

Cell samples were collected and homogenized in Trizol reagent (Qiagen), and RNA extraction and reverse transcription was performed. For PCR amplification of the cDNA fragment coding for targeted genes, sense and antisense primer sequences are listed in Table S2, Supporting Information. Data were normalized to β‐actin levels and were presented as the mean ± SEM. Significance was calculated by unpaired Student's *t* tests.

### Ch‐IP Assay

Ch‐IP assay was performed using the SimpleChIP Kit (Cell Signaling Technology). Approximately 4 × 10^6^ SCLC cells with conditioned treatment for each immunoprecipitation were fixed with 37% formaldehyde. The crosslinked DNA complexes were sheared to lengths of approximately 150–900 base pair fragments and immunoprecipitated with anti‐LEF1, anti‐c‐Myc, anti‐histone H3 (positive control), or IgG (negative) control antibody overnight at 4 °C with rotation. The immunoprecipitated DNA was purified and amplified by PCR. Primers sequences are listed in Table S3, Supporting Information.

### Luciferase Assay

SCLC cells transfected with indicated pcDNA plasmids or pGL3.1 luciferase plasmids by using Lipofectamine 3000 reagent (Invitrogen). After 48 h transfection, cells were subjected to dual luciferase analysis. The results are expressed as a fold induction relative to the cells transfected with the control vector after normalization to Renilla activity.

### Data Collection and Bioinformatics Analysis

The HDAC7 mRNA expression levels and patient follow‐up data across various cancer types were retrieved from TCGA, while the HDAC7 mRNA expression levels in cell lines were obtained from the CCLE database. We measured DEGs between patients with low and high HDAC7 expression. RNA‐sequencing data were obtained from the George et al. cohort^[^ ^33^ ^]^ and GSE60052.^[^ ^34^ ^]^

### Statistical Analysis

Statistical analysis was performed using SPSS version 21.0. The data were subjected to variance analysis (ANOVA), followed by two‐tailed, unpaired Student *t*‐test. Differences with *p* < 0.05 were considered statistically significant. The authors declare that all the other data supporting the findings of this study are available within the article and its Supplementary Information files, and from the corresponding author upon reasonable request.

## Conflict of Interest

The authors declare no conflict of interest.

## Supporting information



Supporting Information

## Data Availability

The data that support the findings of this study are available from the corresponding author upon reasonable request.
