# Supplementary material for: Nuclear to Cytoplasmic Transport Is a Druggable Dependency in HDAC7‐driven Small Cell Lung Cancer
Source: Adv Sci (Weinh). 2025 Jan 30;12(14):2413445. doi: 10.1002/advs.202413445 (PMC11984897; doi:10.1002/advs.202413445)
Supplement: Supplementary file 1 — Supporting Information [file ADVS-12-2413445-s001.docx]

Supporting Information

Nuclear to cytoplasmic transport is a druggable dependency in HDAC7-driven small cell lung cancer

*Tingting Qin^1^, Jingya Wang^1^, Jian Wang^1^, Qingwu Du^1^, Liuchun Wang^1^, Hailin Liu^1^, Wenting Liu^1^, Xueyang Li^1^, Yantao Jiang^1^, Qi Xu^1^, Junjie Yu^1^, Huiyan Liu^1^, Ting Wang^1^, Mengjie Li^1^* and Dingzhi Huang^1^**

**Figure. S1**

We further evaluated the possible correlation between XPO1 and c-Myc expression and found that XPO1 was positively correlated with c-Myc (Spearman r = 0.2680, *P* < 0.0001) **(Figure. S1A)**. Kaplan–Meier analysis suggested that patients with high XPO1/c-Myc had the worst PFS (P = 0.0054) **(Figure. S1B)** and OS (*P* = 0.0085) **(Figure. S1C)**, whereas SCLC patients with low XPO1/c-Myc showed the best outcome. Quantitative analysis indicated that ~72.3% of HDAC7 OE group and ~52.3% NC group exhibited nuclear localization of β-catenin, compared with ~17.6% in HDAC7 KO group **(Figure. S1D)**. The nuclear–cytosolic protein assay confirmed that HDAC7 overexpression promoted β-catenin nuclear import, while HDAC7 KO inhibited β-catenin nuclear import partly **(Figures. S1E and S1F)**. To validate the significance of HDAC7/c-Myc/XPO1 signaling in regulating SCLC proliferation, we investigated the consequences of individual and combined knockouts of c-Myc, XPO1, and c-Myc & XPO1 in NCI-H1688 cells that stably overexpress HDAC7. After knocking out HDAC7, c-Myc, and XPO1, we observed a partial reversal of the enhanced cell growth induced by HDAC7 overexpression **(Figures. S1G, S1H)**. Additionally, there was a further reduction in the levels of c-Myc, XPO1, and ectopic HDAC7 proteins in the HDAC7-overexpressing NCI-H1688 cell lines **(Figure. S1I)**. Notably, compared to the HDAC7-overexpressing (OE) cells treated with selinexor, the c-Myc knockout (KO), XPO1 KO, and simultaneous c-Myc & XPO1 KO groups significantly potentiated the antiproliferative effect of selinexor against HDAC7 OE NCI-H1688 cells **(Figures. S1G, S1H)**. Furthermore, these KO groups inhibited the expression of HDAC7, c-Myc, and XPO1 proteins **(Figure. S1I)**.


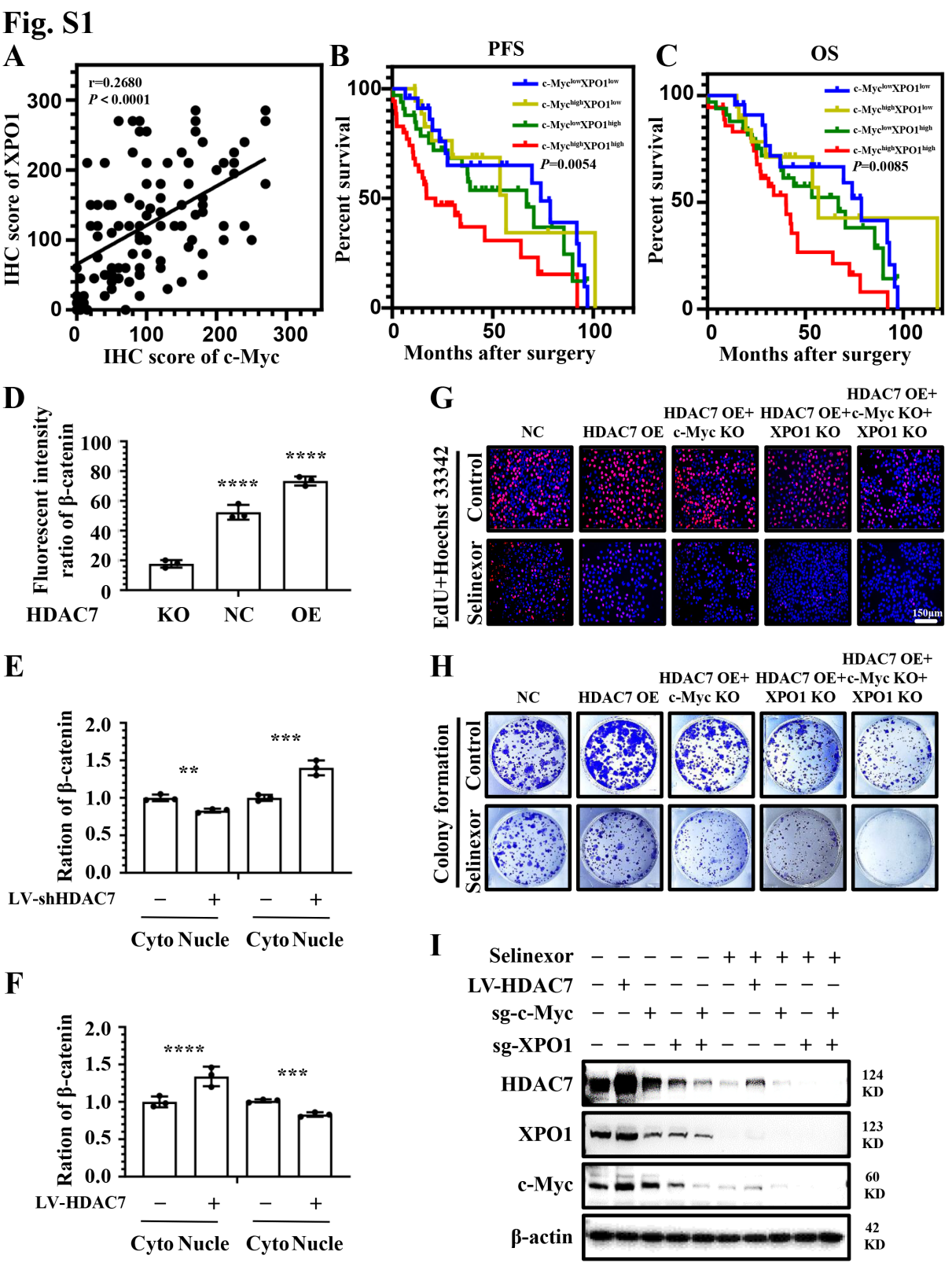


(**A**) Scatterplot showing Spearman’s correlation of IHC score of c-Myc and XPO1 (r = 0.2680, *P* < 0.0001). (**B**) Kaplan–Meier analysis of the association between PFS and the expression of c-Myc and XPO1 in SCLC patients. (**C**) Kaplan–Meier analysis of the association between OS and the expression of c-Myc and XPO1 in SCLC patients. **(D)** The ratio of nuclear catenin fluorescence to total cellular fluorescence. (**E and F**) Densitometry analysis of β-catenin protein levels showen in Figure 5G. Data are mean values ± SD. ***P* < 0.01; ****P* < 0.001; *****P* < 0.0001; one-way ANOVA. (**G**) Typical fluorescent confocal microscopic images of EdU-positive cells in SCLC cell lines with different treatment; Red, Edu positive cells; Blue, nuclei; Scale bar, 150μm. (**H**) Changes in the numbers of colonies in SCLC cell lines with different treatment. (**I**) Changes in HDAC7, XPO1, c-Myc, p21, and p27 protein levels with different treatment.

**Figures. S2-S4 Uncropped data.**

The uncropped illustrations used to prepare the main figures of this manuscript are shown with reference to their specific sub-figures indicated on each panel.


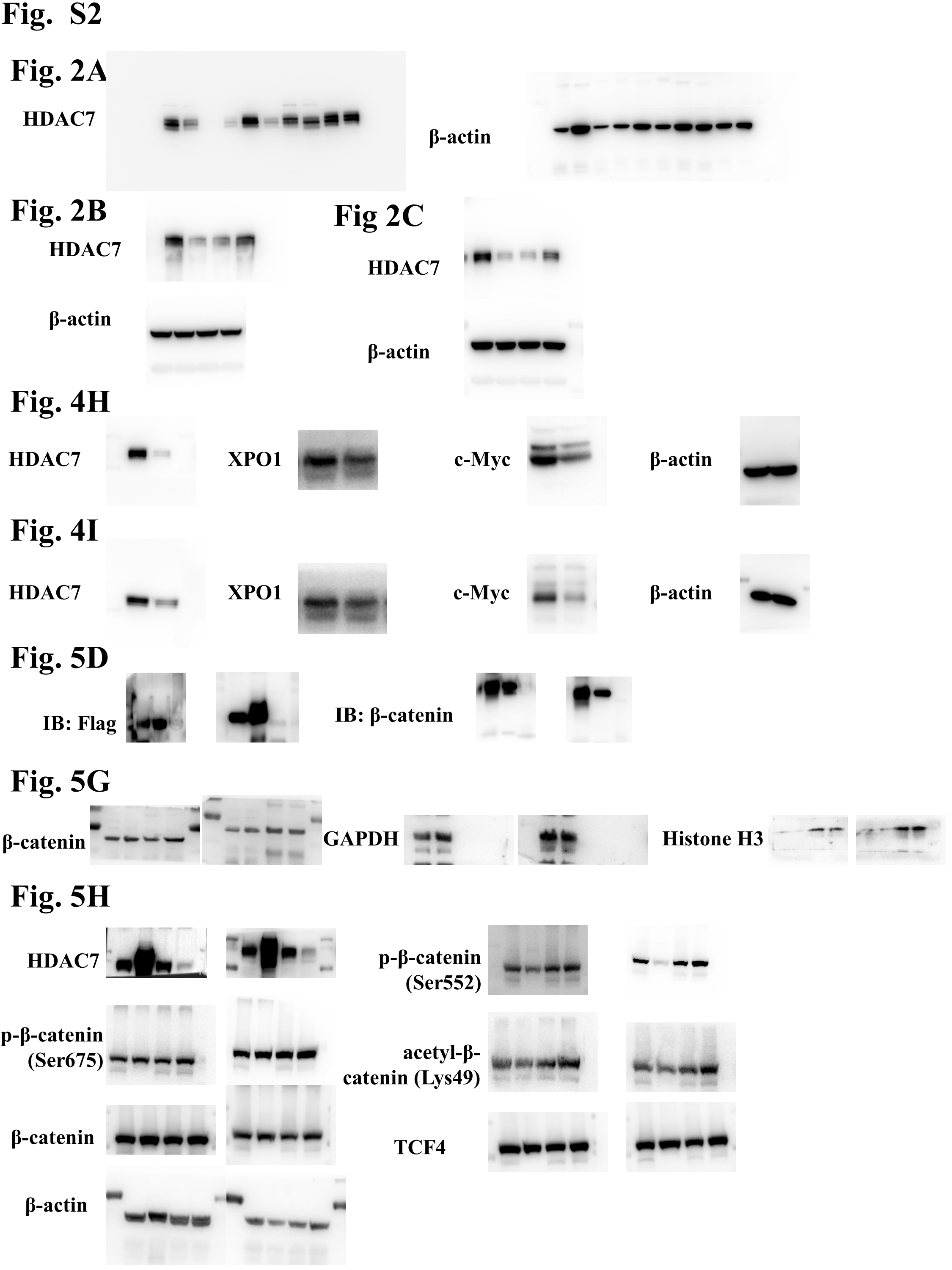


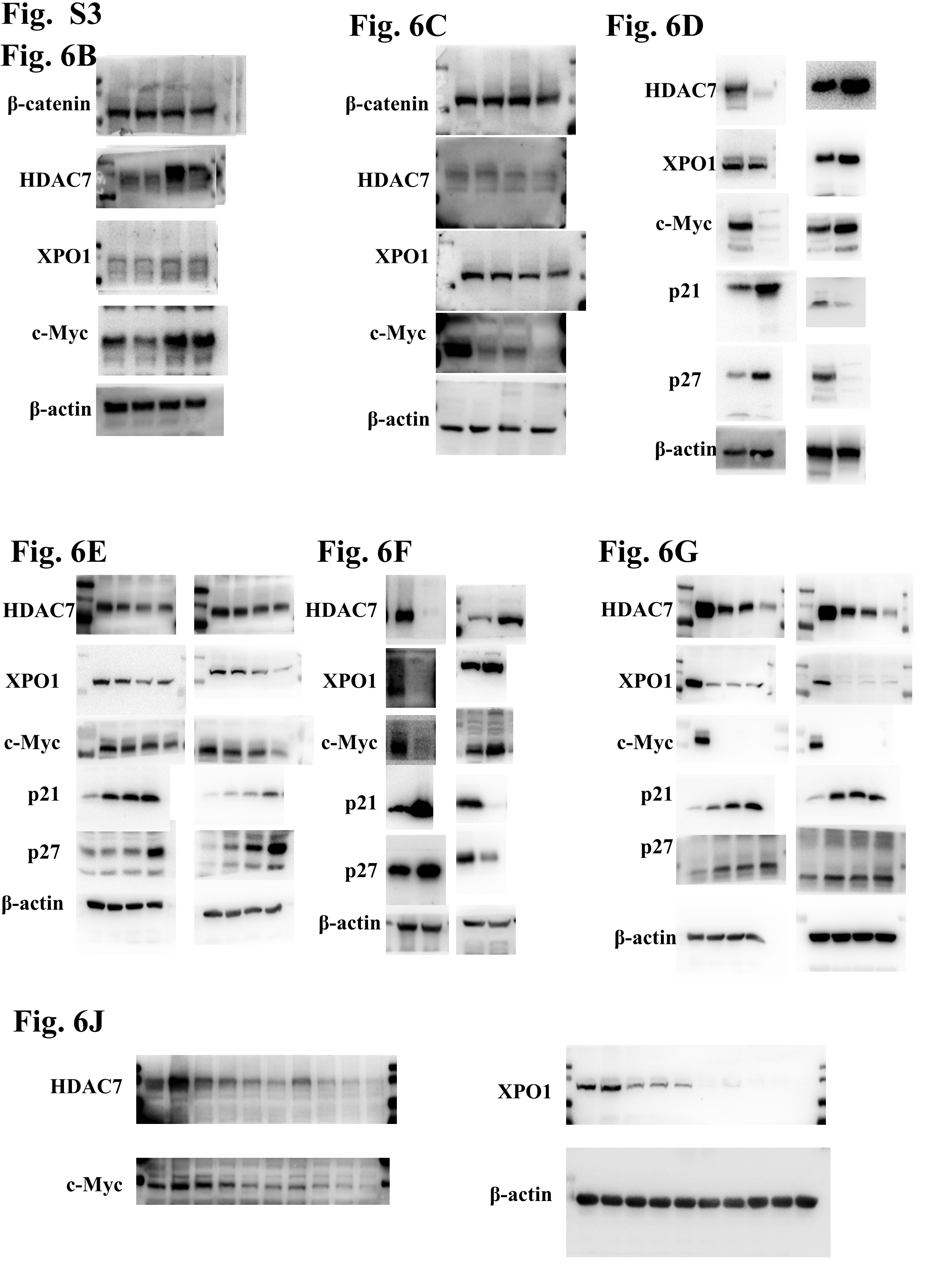


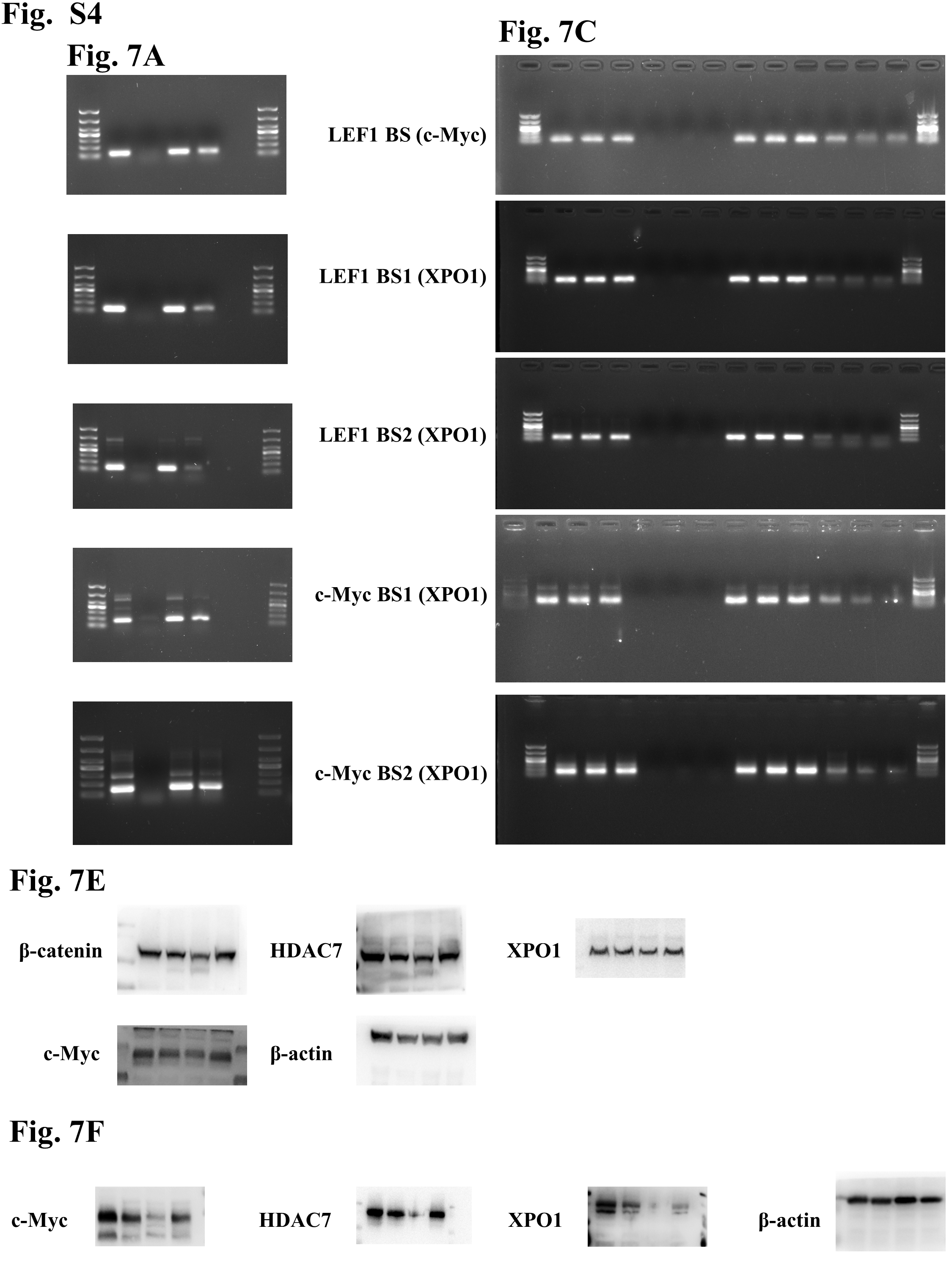


Table S1.

Oligonucleotide sequences used in this study.

| **sgRNA Sequences** | |
| --- | --- |
| sgHDAC7#1 | GATCCGGGTGCACAGTAAATA |
| sg HDAC7#2 | TCACTGACCTCGCCTTCAAAG |
| sg HDAC7#3 | GCTTCTCGTGAGCTAAAGAAT |
| sgβ-catenin#1 | CTAACAGCCGCTTTTCTGTC |
| sgβ-catenin#2 | AGTCCTGTATGAGTGGGAAC |
| sgβ-catenin#3 | TGCAATGACTCGAGCTCAGA |
| sgXPO1#1 | GTGGTGAATTGCTTATACCA |
| sgXPO1#2 | TCGACTCTTGTCCAAGCATC |
| sgXPO1#3 | GCTAACATTGTCATAATTGC |
| sgc-Myc#1 | GCCGTATTTCTACTGCGACG |
| sgc-Myc#2 | CTATGACCTCGACTACGACT |
| sgc-Myc#3 | GTCGAGGTCATAGTTCCTGT |

Table S2.

Oligonucleotide sequences used in this study.

| **Oligonucleotides (Primers)** | |
| --- | --- |
| HDAC7 sense | TCCTGGCACAGCGGATGTTTGT |
| HDAC7 antisense | TGAAGGCGAGGTCAGTGACACT |
| XPO1 sense | CTACATCTGCCTCTCCGTTGCT |
| XPO1 antisense | CCAATACTTCCTCTGGTTTAGCC |
| Ran sense | CCACCAGAAGTTGTCATGGACC |
| Ran antisense | CTCCAGCTTCATTCTCACAGGTC |
| RanBP1 sense | ACCATGACCCTCAGTTTGAGCC |
| RanBP1 antisense | AGTGCCTCGCTCCTTCCATTCT |
| EIF4E sense | ATGCCTGGCTGTGACTACTCAC |
| EIF4E antisense | GAGGTCACTTCGTCTCTGCTGT |
| c-Myc sense | CCTGGTGCTCCATGAGGAGAC |
| c-Myc antisense | CAGACTCTGACCTTTTGCCAGG |
| β-actin sense | CACCATTGGCAATGAGCGGTTC |
| β-actin antisense | AGGTCTTTGCGGATGTCCACGT |

Table S3.

Oligonucleotide sequences used in this study.

| **Oligonucleotides (Primers)** | |
| --- | --- |
| LEF1 MYC site1(119bp)-sense | TCCCATATTCTCCCGTCTA |
| LEF1 MYC site1(119bp)-antisense | TTTCTCAGCCAGGTTTCA |
| LEF1 XPO1 site1(130bp)-sense | GAAGTGCAGCTAGGGTAAA |
| LEF1 XPO1 site1(130bp)-antisense | AACATTGTAATCCCAAGTGA |
| LEF1 XPO1 site2(132bp)-sense | AAGTCGCAGTTTCCAAG |
| LEF1 XPO1 site2(132bp)-antisense | AGTTTACATCGTATAAGGTATT |
| MYC XPO1 site1(136bp)-sense | TGCGCTGTCAAACCAA |
| MYC XPO1 site1(136bp)-antisense | CAGGGCGTGCATTCTT |
| MYC XPO1 site2(154bp)-sense | GATTTGGAGGACGCTTGG |
| MYC XPO1 site2(154bp)-antisense | CTGGTGCCCTTGATCTGC |
| MYC XPO1 site3(178bp)-sense | CGCTTAGCCTCCCGAGTA |
| MYC XPO1 site3(178bp)-antisense | ATGGTGGTTCATGCCTTT |

Table S4. Clinical characteristics of 110 patients with Small Cell lung Cancer.

| **Characteristics** | **ALL, n (%)** |
| --- | --- |
| **Gender** |  |
| Male | 86 (78.2) |
| Female | 24 (21.8) |
| **Age** |  |
| ＜60 | 49 (44.5) |
| ≥60 | 61 (55.5) |
| **Smoking history** |  |
| Yes | 93 (84.5) |
| No | 17 (15.5) |
| **TNM Stage** |  |
| I-II | 92 (83.6) |
| III-IV | 18 (16.4) |
| **HDAC7** |  |
| H-socre＜120 | 53 (48.2) |
| H-socre≥120 | 57 (51.8) |
| **XPO1** |  |
| H-socre ＜120 | 49 (44.5) |
| H-socre≥120 | 61 (55.5) |
| **c-Myc** |  |
| H-socre ＜90 | 60 (54.5) |
| H-socre ≥90 | 50 (45.5) |

**Table S5. Association of HDAC7/XPO1/c-Myc expression with clinical characteristics of patients with SCLC.**

| **Characteristics** | **Total**  **(n=110)** | **HDAC7**  **low, n (%)** | **HDAC7 high, n (%)** | **p-Value** | **XPO1**  **low, n (%)** | **XPO1**  **high, n (%)** | **p-Value** | **c-Myc**  **low, n (%)** | **c-Myc high, n (%)** | **p-Value** |
| --- | --- | --- | --- | --- | --- | --- | --- | --- | --- | --- |
| **Gender** |  |  |  | 0.795 |  |  | 0.886 |  |  | 0.332 |
| Male | 86 | 42 (79.2) | 44 (77.2) |  | 38 (77.6) | 48 (78.7) |  | 37 (74.0) | 49 (81.7) |  |
| Female | 24 | 11 (20.8) | 13 (22.8) |  | 11 (22.4) | 13 (21.3) |  | 13 (26.0) | 11 (18.3) |  |
| **Age** |  |  |  | 0.316 |  |  | 0.481 |  |  | 0.624 |
| ＜60 | 49 | 21 (39.6) | 28 (57.1) |  | 20 (40.8) | 29 (47.5) |  | 21 (42.0) | 28 (46.7) |  |
| ≥60 | 61 | 32 (60.4) | 29 (50.9) |  | 29 (59.2) | 32 (52.5) |  | 29 (58.0) | 32 (53.3) |  |
| **Smoking history** |  |  |  | 0.920 |  |  | 0.761 |  |  | 0.885 |
| Yes | 93 | 45 (84.9) | 48 (84.2) |  | 42 (85.7) | 51 (83.6) |  | 42 (84.0) | 51 (85.0) |  |
| No | 17 | 8 (15.1) | 9 (15.8) |  | 7 (14.3) | 10 (16.4) |  | 8 (16.0) | 9 (15.0) |  |
| **TNM Stage** |  |  |  | 0.042 |  |  | 0.053 |  |  | 0.049 |
| Ⅰ-II | 92 | 42 (91.3) | 50 (78.1) |  | 39 (86.7) | 53 (81.5) |  | 39 (88.6) | 53 (80.3) |  |
| ⅡI-IV | 18 | 4 (8.7) | 14 (21.9) |  | 6 (13.3) | 12 (18.5) |  | 5 (11.7) | 13 (19.7) |  |
| **T stage** |  |  |  | 0.169 |  |  | 0.220 |  |  | 0.186 |
| T1 | 18 | 11 (20.8） | 7 (12.3) |  | 10 (20.4） | 8 (13.1) |  | 10 (20.0) | 8 (13.3) |  |
| T2 | 33 | 11 (20.8） | 22 (38.6) |  | 10 (20.4） | 23 (37.7) |  | 10 (20.0) | 23 (38.3) |  |
| T3  T2T4 | 56 | 30 (56.6） | 26 (45.6) |  | 28 (50.0） | 28 (50.0) |  | 28 (56.0) | 28 (46.7) |  |
| T4 | 3 | 1 (1.9） | 2 (3.5) |  | 1 (2.0） | 2 (3.3) |  | 2 (4.0) | 1 (1.7) |  |
| **Lymphatic invasion** |  |  |  | 0.0403 |  |  | 0.043 |  |  | 0.0317 |
| N0 | 64 | 33 (62.3） | 31 (54.4) |  | 31 (63.3） | 33 (54.1) |  | 39 (72.2） | 25 (64.8) |  |
| N1-N3 | 46 | 20 (37.7） | 26 (45.6) |  | 18 (36.7） | 28 (45.9) |  | 15 (27.8） | 31 (35.2) |  |
| **Distant metastasis** |  |  |  | 0.624 |  |  | 0.488 |  |  | 0.154 |
| No | 103 | 49 (92.5） | 54 (94.7) |  | 45 (91.8) | 58 (95.1) |  | 45 (90.0) | 58 (96.7) |  |
| Yes | 7 | 4 (7.5） | 3 (5.3) |  | 4 (8.2) | 3 (4.9) |  | 5 (10.0) | 2 (3.3) |  |
| **Postoperative therapy** |  |  |  | 0.559 |  |  | 0.500 |  |  | 0.976 |
| Treatment | 89 | 44 (93.6) | 45 (86.5) |  | 40 (93.0) | 49 (87.5) |  | 39 (88.6) | 50 (90.9) |  |
| Non-treatment | 10 | 3 (6.4) | 7 (13.5) |  | 3 (7.0) | 7 (12.5) |  | 5 (11.4) | 5 (9.1) |  |

Table S6.

Univariate and multivariate cox analysis of factors for PFS and OS in patients with SCLC.

| **Univariate cox analysis** | | | | | | |
| --- | --- | --- | --- | --- | --- | --- |
|  | **Overall survival** | | | **Progression-free survival** | | |
| **Variable** | **HR** | **95% CI** | **p value** | **HR** | **95% CI** | **p value** |
| Gender (female vs male) | 0.823 | 0.417-1.623 | 0.573 | 0.792 | 0.398-1.575 | 0.506 |
| Age (<60 VS. ≥60) | 0.918 | 0.554-1.522 | 0.740 | 0.909 | 0.548-1.510 | 0.714 |
| Smoking history (no vs. yes) | 1.220 | 0.585-2.541 | 0.596 | 1.180 | 0.561-2.483 | 0.663 |
| T factor (T ≤2 VS. T >2) | 0.794 | 0.471-1.340 | 0.388 | 0.682 | 0.401-1.160 | 0.158 |
| Lymphatic metastasis (N0 VS. N1-N3) | 1.653 | 1.004-2.724 | **0.048** | 1.458 | 0.875-2.430 | 0.148 |
| Distant metastasis (no vs. yes) | 1.392 | 0.542-3.576 | 0.492 | 2.115 | 0.789-5.670 | 0.137 |
| TNM Stage (Ⅰ/Ⅱ vs. Ⅲ) | 0.382 | 0.164-0.887 | **0.025** | 0.332 | 0.139-0.793 | **0.013** |
| HDAC7 expression (High vs. Low) | 0.274 | 0.101-0.742 | **0.011** | 0.353 | 0.139-0.894 | **0.028** |
| XPO1 expression (High vs. Low) | 0.299 | 0.120-0.744 | **0.010** | 0.372 | 0.155-0.893 | **0.027** |
| c-Myc expression (High vs. Low) | 5.343 | 2.013-14.182 | **0.001** | 3.417 | 1.447-8.067 | **0.005** |
| **Multivariate cox analysis** | | | | | | |
| Lymphatic metastasis (N0 VS. N1-N3) | 0.723 | 0.436-1.198 | 0.208 | 0.807 | 0.482-1.350 | 0.414 |
| TNM Stage (Ⅰ/Ⅱ vs. Ⅲ) | 2.151 | 0.950-4.868 | 0.066 | 2.077 | 0.923-4.675 | 0.078 |
| HDAC7 expression (High vs. Low) | 0.341 | 0.125-0.926 | **0.035** | 0.415 | 0.163-1.057 | 0.065 |
| XPO1 expression (High vs. Low) | 0.266 | 0.097-0.731 | **0.010** | 0.347 | 0.137-0.878 | **0.025** |
| c-Myc expression (High vs. Low) | 5.634 | 2.072-15.318 | **0.001** | 3.566 | 1.462-8.698 | **0.005** |
